# Prothymosin α and a prothymosin α-derived peptide enhance T_H_1-type immune responses against defined HER-2/neu epitopes

**DOI:** 10.1186/1471-2172-14-43

**Published:** 2013-09-22

**Authors:** Kyriaki Ioannou, Evelyna Derhovanessian, Eleni Tsakiri, Pinelopi Samara, Hubert Kalbacher, Wolfgang Voelter, Ioannis P Trougakos, Graham Pawelec, Ourania E Tsitsilonis

**Affiliations:** 1 Department of Animal and Human Physiology, Faculty of Biology, University of Athens, Athens 15784, Greece; 2 Department of Internal Medicine II, Centre for Medical Research, University of Tübingen, Tübingen 72072, Germany; 3 Department of Cell Biology and Biophysics, Faculty of Biology, University of Athens, Athens 15784, Greece; 4 Interfakultäres Institut für Biochemie, University of Tübingen, Tübingen 72072, Germany

**Keywords:** Prothymosin alpha, Immunoreactive peptide, Dendritic cells, T_H_1 immune responses, TLR-4, Adjuvant, HER-2/neu peptides

## Abstract

**Background:**

Active cancer immunotherapies are beginning to yield clinical benefit, especially those using peptide-pulsed dendritic cells (DCs). Different adjuvants, including Toll-like receptor (TLR) agonists, commonly co-administered to cancer patients as part of a DC-based vaccine, are being widely tested in the clinical setting. However, endogenous DCs in tumor-bearing individuals are often dysfunctional, suggesting that *ex vivo* educated DCs might be superior inducers of anti-tumor immune responses. We have previously shown that prothymosin alpha (proTα) and its immunoreactive decapeptide proTα(100–109) induce the maturation of human DCs *in vitro.* The aim of this study was to investigate whether proTα- or proTα(100–109)-matured DCs are functionally competent and to provide preliminary evidence for the mode of action of these agents.

**Results:**

Monocyte-derived DCs matured *in vitro* with proTα or proTα(100–109) express co-stimulatory molecules and secrete pro-inflammatory cytokines. ProTα- and proTα(100–109)-matured DCs pulsed with HER-2/neu peptides induce T_H_1-type immune responses, prime autologous naïve CD8-positive (+) T cells to lyse targets expressing the HER-2/neu epitopes and to express a polyfunctional profile, and stimulate CD4+ T cell proliferation in an HER-2/neu peptide-dependent manner. DC maturation induced by proTα and proTα(100–109) is likely mediated *via* TLR-4, as shown by assessing TLR-4 surface expression and the levels of the intracellular adaptor molecules TIRAP, MyD88 and TRIF.

**Conclusions:**

Our results suggest that proTα and proTα(100–109) induce both the maturation and the T cell stimulatory capacity of DCs. Although further studies are needed, evidence for a possible proTα and proTα(100–109) interaction with TLR-4 is provided. The initial hypothesis that proTα and the proTα-derived immunoactive decapeptide act as “alarmins”, provides a rationale for their eventual use as adjuvants in DC-based anti-cancer immunotherapy.

## Background

Anti-cancer vaccines are designed to break tolerance to self and stimulate strong and durable anti-tumor immunity. Administering defined tumor-derived epitopes to cancer patients for the activation of helper and cytotoxic T cells has been shown to enhance anti-cancer immune responses *in vivo* and in some cases to lead to objective clinical responses [1-3]. To optimize the efficacy of peptide-based anti-cancer vaccines, combinatorial approaches stimulating both innate and adaptive immunity are now being clinically evaluated [4,5]. Mature dendritic cells (DCs) are key players for eliciting such responses, as they present antigens to T cells and provide the necessary co-stimulatory signals and cytokines favoring the efficient activation of tumor-reactive immune cells [6,7]. DC maturation can be induced *in vivo* upon admixing and co-administering immunogenic peptides with adjuvants, but to date this strategy has been proven successful only when vaccinating against common pathogens [8]. In cancer patients, the presence of tumor-associated suppressive factors impairs endogenous DC functions [9], a condition that can be bypassed only by the adoptive transfer of *ex vivo* matured immunocompetent DCs [10,11].

Adjuvants comprise, among others, Toll-like receptor (TLR) agonists, the majority of which reportedly promotes DC maturation [12]. A subcategory thereof are molecules with so-called pathogen-associated molecular patterns (PAMPs), such as CpG oligodeoxynucleotides that signal through TLR-9 [13], poly-I:C ligating TLR-3 [14], imiquimod, a TLR-7 agonist [15] and monophosphoryl lipid A, a TLR-4 agonist [16]. A second group consists of molecules possessing damage-associated molecular patterns (DAMPs) or “alarmins”. High mobility group box 1 (HMGB1) protein and heat shock protein (HSP) 90 are notable examples of DAMPs. Both proteins are strictly intracellular under normal physiological conditions, but when excreted eg. from damaged cells, signal through TLR-4, sensitize DCs and promote adaptive immune responses [17]. This functional dualism, in and out of the cell, also characterizes prothymosin alpha (proTα).

In normal living cells, proTα is localized in the nucleus where it controls the cell cycle and promotes cell proliferation. Released from dead cells, extracellular proTα acquires multi-functional immunomodulatory properties [18]. We and others have previously shown that proTα upregulates the expression of IRAK-4 in human monocytes [19], ligates TLR-4 on murine macrophages and signals through MyD88-dependent and independent pathways [20]. Similar to its immunoreactive decapeptide proTα(100–109) [21], it upregulates the expression of HLA-DR [22], CD80, CD83 and CD86 and promotes maturation of human DCs *in vitro*[23].

Here, we show that DCs matured *ex vivo* in the presence of proTα or proTα(100–109) are not only phenotypically but also functionally competent, secrete pro-inflammatory cytokines and induce T_H_1-type immune responses in the presence of tumor-associated immunogenic epitopes of the oncoprotein HER-2/neu. DCs matured with proTα or proTα(100–109) prime naïve CD8-positive (+) T cells to exert HER-2/neu peptide-specific cytotoxicity and CD4+ T cells to proliferate in a peptide-dependent manner. Finally, we provide preliminary evidence suggesting that both proTα and its decapeptide proTα(100–109) likely signal *via* TLR-4 in human DCs.

## Results

### Phenotype of and cytokine production by proTα- or proTα(100–109)-matured DCs

**Figure 1:**
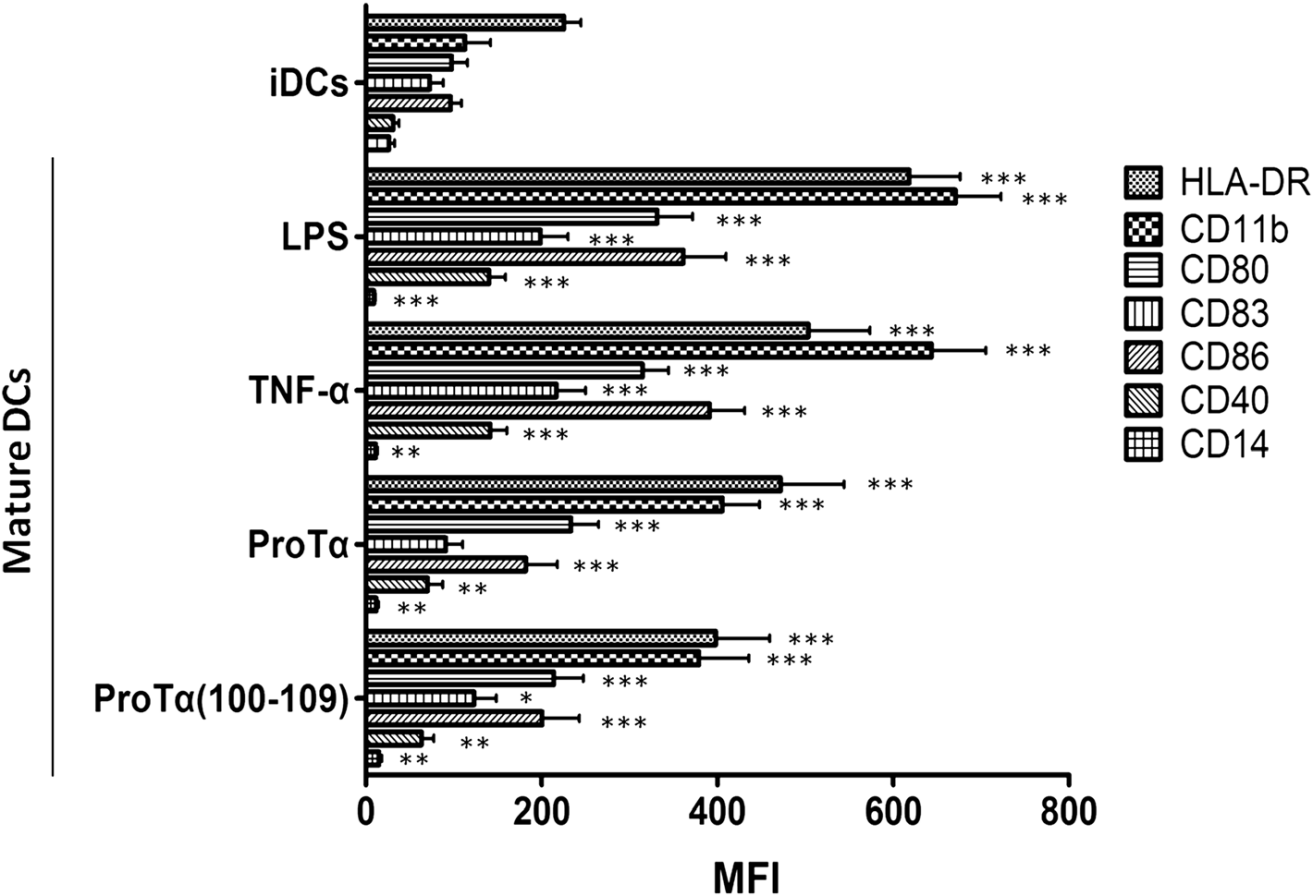
**ProTα or proTα(100–109) induce DC maturation.** Monocytes were differentiated to immature DCs (iDCs) upon 5-day incubation with GM-CSF and IL-4, followed by 48 h exposure to LPS, TNF-α, proTα or proTα(100–109). Expression of surface HLA-DR, CD11b, CD80, CD83, CD86, CD40 and CD14 on iDCs and mature DCs are shown as mean fluorescence intensity (MFI) ± SD from 5 donors. * *p* < 0.05; ** *p* < 0.01; *** *p* < 0.001, compared to iDCs.

We have previously shown that proTα and proTα(100–109) efficiently mature human DCs *in vitro*, as indicated by the induction of surface expression of established DC-markers to levels comparable to those induced by lipopolysacharide (LPS) [23] or tumor necrosis factor (TNF)-α (this report). As shown in Figure 1, LPS-matured DCs significantly upregulated the expression of HLA-DR, CD11b, CD80, CD83, CD86 and CD40, to levels comparable to TNF-α-matured DCs (*p* < 0.001 compared to iDCs for all values). Similarly, both agents caused a reduction of CD14 expression. In agreement with our previous study [23], iDCs matured with either proTα or its decapeptide presented a similar phenotype to LPS- or TNF-α-matured DCs, upregulating the expression of all co-stimulatory molecules (*p* < 0.05-0.001, compared to iDCs; Figure 1) and downregulating CD14 (*p* < 0.01, compared to iDCs).

**Figure 2:**
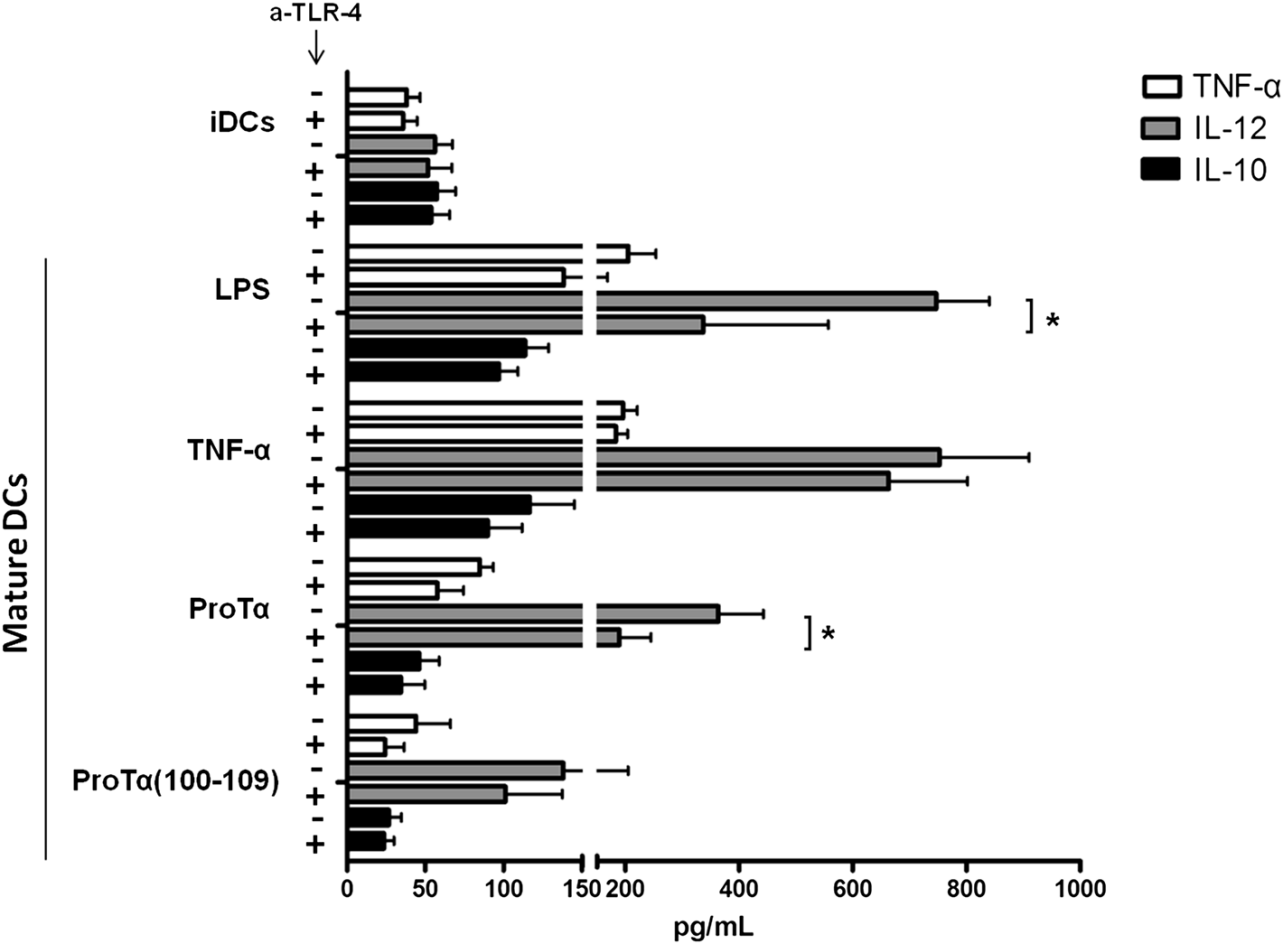
**ProTα- or proTα(100–109)-matured DCs secrete pro-inflammatory cytokines.** Culture supernatants from iDCs and DCs matured with LPS, TNF-α, proTα or proTα(100–109) for 48 h were assessed for their TNF-α, IL-12 and IL-10 content. iDCs were treated (+) or not (−) with an anti-TLR-4 mAb (a-TLR-4) for 1 h prior to their maturation. Data are given as mean values ± SDs from 3 donors. * *p* < 0.05.

In conjunction with their phenotype, functionally competent mature DCs secrete pro- and anti-inflammatory cytokines [6]. Therefore, we assessed the production of TNF-α, interleukin (IL)-12 and IL-10 from iDCs and mature DCs and determined the IL-12:IL-10 ratio. We present these data because high TNF-α levels, as well as a balance between IL-12:IL-10 in favor of IL-12 have been proposed to promote T_H_1-polarization [24]. As shown in Figure 2, iDCs produced low amounts of all three cytokines, whereas mature DC supernatants collected 48 h after addition of LPS or TNF-α were rich in TNF-α and IL-12. Compared to iDCs, higher TNF-α and IL-12 levels were also found in supernatants of cultures of DCs matured with proTα and proTα(100–109). Although the absolute concentrations of cytokines varied among the differentially matured DCs, their overall cytokine-production patterns were comparable. Most importantly, the mean IL-12:IL-10 ratios were similar (6.61, 6.45, 7.89 and 5.18, for LPS-, TNF-α-, proTα- and proTα(100–109)-matured DCs, respectively). These data suggest that the peptides bias immunoreactivity towards a pro-inflammatory T_H_1-type of response.

Finally, in the presence of a blocking antibody against TLR-4 (a-TLR-4; Figure 2), lower amounts of cytokines were secreted by LPS-, proTα- and proTα(100–109)-matured DCs, but not TNF-α-matured DCs. Notably, a-TLR-4 reduced the levels of LPS- and proTα-induced IL-12 production by 55 and 47%, respectively (*p* < 0.05), implying that IL-12 production by LPS- and proTα-matured DCs is at least partially, TLR-4-dependent [25].

### ProTα and proTα(100–109) lead to T_H_1-polarized tumor peptide-reactive immune response

As optimally matured DCs prime antigen-specific CD4+ and CD8+ T cell activation and proliferation of naive T cells [7], we next assessed whether proTα- and proTα(100–109)-matured DCs are functionally competent, i.e., induce *in vitro* the selective expansion of tumor antigen-specific T cells.

**Figure 3:**
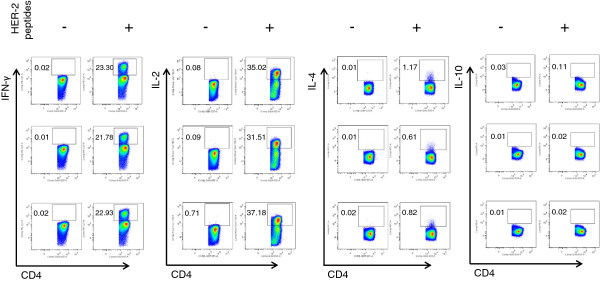
**T cells stimulated with proTα- or proTα(100–109)-matured HER-2/neu-peptide-pulsed autologous DCs produce T**_**H**_**1-type cytokines.** Monocytes from HLA-A2+/DR4+ donors were differentiated to iDCs, matured for 48 h with TNF-α, proTα or proTα(100–109), pulsed with HER-2(9_369_) and HER-2(15_776_) epitopes and used to stimulate autologous T cells. Recovered CD4+ T cells were analyzed for intracellular production of IFN-γ, IL-2, IL-4 and IL-10, in the absence (−) or presence (+) of the HER-2/neu peptides. First row, DCs matured with TNF-α; middle row, DCs matured with proTα; bottom row, DCs matured with proTα (100–109). Numbers indicate percentage of positive cells for each cytokine on gated CD4+ T cells. Shown data are from one representative donor of 5 tested.

Monocyte-derived DCs matured for 48 h with proTα, proTα(100–109) or TNF-α (used as a conventional DC maturation agent) were pulsed with the HLA-A2 and HLA-DR4-restricted HER-2/neu(369–377) [HER-2(9_369_)] and HER-2/neu(776–790) [HER-2(15_776_)] epitopes, and used to prime autologous naïve T cells isolated from the peripheral blood of HLA-A2+/DR4+ healthy donors. T cells were restimulated twice, at weekly intervals, with similarly matured autologous DCs. Twelve hours after the third stimulation their production of TNF-α, interferon (IFN)-γ, IL-2, IL-4, IL-10 and IL-17 was analysed. Figure 3 shows the percentages of IFN-γ+, IL-2+, IL-4+ and IL-10+ CD4+ T cells from one representative donor of 5 tested with similar results (Additional file 1: Table S1A). In the presence of unpulsed TNF-α-matured DCs, only a low percentage of CD4+ T cells produced IFN-γ (0.02%), which was significantly increased (23.30%) in the presence of the HER-2/neu peptides. An analogous increase in the percentage of IFN-γ-producing cells was also recorded in CD4+ T cells stimulated with proTα- or proTα(100–109)-matured DCs in the presence of the same peptides (21.78% and 22.93%, respectively, compared to 0.01% and 0.02% in the absence of HER-2/neu peptides; Figure 3). The percentages of IL-2-producing CD4+ T cells in all groups were also significantly enhanced upon stimulation with HER-2/neu peptide-pulsed DCs (35.02% for TNF-α-, 31.51% for proTα- and 37.18% for proTα(100–109)-matured DCs, compared to 0.08%, 0.09% and 0.71% of the respective unpulsed groups; Figure 3). A similar enhancement was also seen for TNF-α-producing CD4+ T cells (Additional file 1: Table S1A). In contrast, production of IL-4 and IL-10 was only marginally increased when CD4+ T cells were stimulated with HER-2/neu peptide-pulsed DCs, regardless of the factor used for DC maturation (Figure 3). Specifically, the percentage of CD4+ T cells producing IL-4 was increased from 0.01% (without peptides) to 1.17% among T cells stimulated with peptide-pulsed TNF-α-matured DCs, from 0.01% to 0.61% in the proαα- and from 0.02% to 0.82% in the proTα(100–109)-matured DC groups. A minor enhancement was also recorded for IL-10 production; IL-10+ cells increased from 0.03% to 0.11% in the TNF-α-, from 0.01% to 0.02% in the proαα- and from 0.01% to 0.02% in the proTα(100–109)-matured DC cultures without and with HER-2/neu peptides, respectively. IL-17 production exhibited a similar pattern of marginal increase in CD4+ T cells stimulated in the presence of all matured antigen-pulsed DCs (Additional file 1: Table S1A). These data suggest that proTα- and proTα(100–109)-matured DCs are immunocompetent and in the presence of specific tumor antigenic epitopes, favor the *in vitro* production of pro-inflammatory (IFN-γ, IL-2, TNF-α), rather than anti-inflammatory cytokines (IL-4, IL-10) and IL-17 by CD4+ T cells, inducing T_H_1-type immune responses.

### ProTα- and proTα(100–109)-matured DCs stimulate tumor peptide-specific CD8+ T cell responses

Cell-mediated immunity requires initial collaboration between T_H_1 CD4+ and CD8+ T cells [26]. Thus, we next investigated whether proTα- and proTα(100–109)-matured DCs can elicit tumor peptide-specific cytotoxic T cell immune responses.

**Figure 4:**
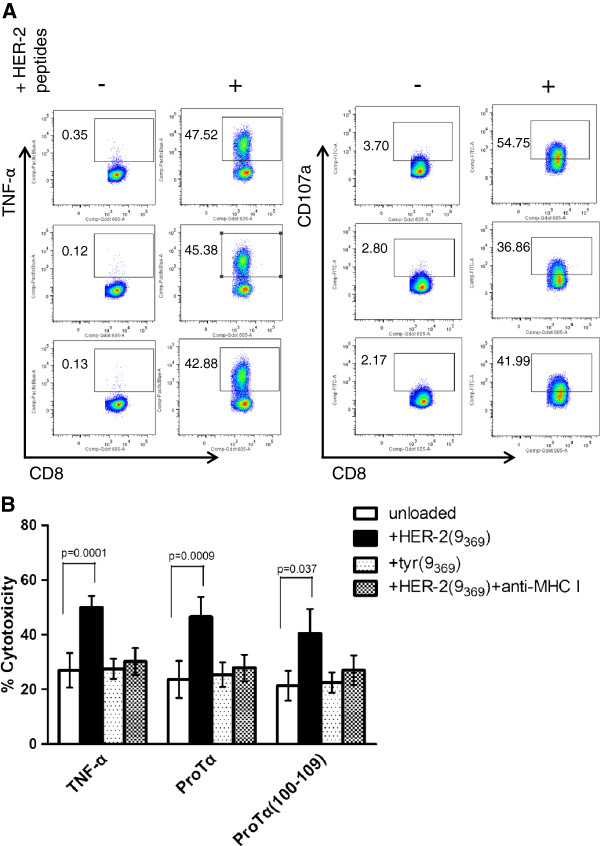
**T cells stimulated with proTα- or proTα(100–109)-matured DCs are cytotoxic in the presence of HER-2(9**_**369**_**). (A)** Intracellular production of TNF-α (left) and surface expression of CD107a (right) in CD8+ T cells stimulated with DCs matured for 48 h with TNF-α (first row), proTα (middle row) or proTα(100–109) (bottom row), in the absence (−) or presence (+) of the HER-2/neu peptides. Numbers indicate percentages of positive cells on gated CD8+ T cells. Shown are data from one representative experiment of 5 performed. **(B)** T cells stimulated with TNF-α-, proTα- or proTα(100–109)-matured DCs, were used as effectors (E) against unloaded, HER-2(9_369_)- and tyr(9_369_)-loaded T2 targets (T). Where indicated, mAb to MHC class I molecules was added throughout the culture period at a final concentration of 5 μg/mL. In all assays the E:T ratio was 10:1. Data represent mean % cytotoxicity ± SD from 2–5 donors.

CD8+ T cells recovered from the same stimulation cultures as aforementioned were assessed for the intracellular production of TNF-α. As shown in Figure 4A, they also exhibited a similar pattern of enhanced cytokine production in the presence of HER-2/neu peptides as did CD4+ T cells. The percentage of TNF-α+ cells was increased from 0.35% (unpulsed) to 47.52% (pulsed) when T cells were stimulated with TNF-α-matured DCs, and from 0.12% to 45.38% for proTα- and from 0.13% to 42.88% for proTα(100–109)-matured DCs (Figure 4A). In addition and in accordance with the results recorded for CD4+ T cells, IL-2- and IFN-γ-producing CD8+ T cells were also increased in the presence of peptide-pulsed DCs in the cultures, whereas differences in the percentages of IL-10-producing CD8+ T cells were only marginal (Additional file 1: Table S1A).

The same cells were assessed for the expression of CD107a, as a surrogate marker for cytotoxicity [27]. In the absence of HER-2(9_369_), a low percentage of CD8+ T cells stimulated with TNF-α-matured DCs expressed CD107a (3.70%; Figure 4A), which increased when cells were stimulated with HER-2(9_369_)-pulsed DCs (54.75%). Similar CD107a upregulation was observed in CD8+ T cells stimulated with proTα- and proTα(100–109)-matured HER-2(9_369_)-pulsed DCs (36.86% and 41.99%, respectively, compared to 2.80% and 2.17% of the unpulsed groups; Figure 4A). Since TNF-α mediates target cell damage and CD107a-expressing CD8+ T cells are cytotoxic [27], our results suggest that proTα- and proTα(100–109)-matured DCs efficiently activate CD8+ cytotoxic T cells, which were able to kill targets presenting the immunogenic epitope *versus* which they were primed.

Cytotoxic activity was verified by using ^51^Cr-labeled HLA-A2+ T2 cells loaded with HER-2(9_369_) or an irrelevant epitope, tyrosinase(369–377) [tyr(9_369_)]. CD8+ T cells thrice stimulated with peptide-pulsed TNF-α-, proTα- or proTα(100–109)-matured DCs were coincubated with these peptide-loaded T2 targets. The results showed that CD8+ T cell mean cytotoxicity against non-peptide loaded T2 targets did not exceed 30% in any group (26.9% for TNF-α, 23.7% for proTα- and 21.4% for proTα(100–109)-matured DCs; Figure 4B), whereas HER-2(9_369_)-loaded T2 targets were lysed twice as efficiently by CD8+ T cells recovered from all stimulation cultures (49.9% for TNF-α-, 46.6% for proTα- and 40.4% for proTα(100–109)-matured DCs; Figure 4B). Cytotoxicity against T2 targets loaded with tyr(9_369_) was low and in no instance exceeded 30%. These cytotoxic responses were significantly decreased by monoclonal antibody (mAb) to MHC class I molecules, suggesting that the CD8+ T cells generated by our stimulation protocol are MHC class I-restricted and HER-2(9_369_)-specific (Figure 4B).

### Polyfunctionality of HER-2(9_369_)–specific CD8+ T cells

**Figure 5:**
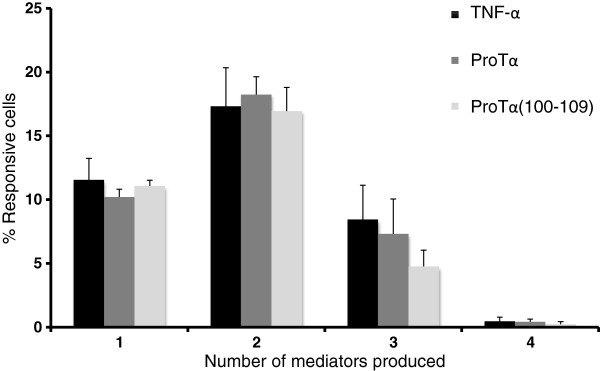
**HER-2(9**_**369**_**)-specific T cells, stimulated with proTα- or proTα(100–109)-matured DCs, are polyfunctional*.*** The proportion of cells producing IFN-γ, TNF-α, IL-2 or CD107a was determined in total responsive CD8+ T cells recovered from cultures stimulated with DCs matured with TNF-α, proTα or proTα(100–109). Mean ± SE of data obtained from 3 different donors are shown.

Based on previous studies associating T cell polyfunctionality with high IFN-γ production and the quality of the elicited responses [28,29], we carried out a functional analysis of the HER-2(9_369_)-specific CD8+ T cells generated in these experiments. Using FlowJo software, we analyzed their ability to produce effector cytokines (IFN-γ, TNF-α and IL-2) and to degranulate (expression of CD107a). Quantifying the fraction of the responsive CD8+ T cells producing any one (1+), any two (2+), any three (3+) or all four (4+) mediators, we observed that approximately a mean ~16% of the responsive CD8+ T cells were 2+ cells, regardless of the agent used to mature the DCs that stimulated them (16.26% for TNF-α; 16.92% for proTα; 15.95% for proTα(100–109); Figure 5). In all experimental groups, 3+ cells were also detected in increased percentages (8.13% for TNF-α; 7.03% for proTα; 4.34% for proTα(100–109)). In contrast, very few 4+ cells were detected under any conditions. Taken together, these data suggest that proTα- or proTα(100–109)-matured DCs were able to induce polyfunctional (2+, 3+) CD8+ peptide-specific T cell responses at least as well as TNF-α-matured DCs.

### T cells stimulated with proTα- or proTα(100–109)-matured DCs proliferate in response to the HER-2(15_776_) epitope

T cell proliferation was assessed by the incorporation of ^3^H-thymidine. HER-2(15_776_)-sensitized T cells coincubated with HER-2(15_776_)-, tyrosinase(448–462) [tyr(15_448_)]-pulsed or unpulsed DCs, specifically proliferated in response only to the HER-2/neu epitope (Table 1). ProTα- and proTα(100–109)-matured DCs showed relatively high mean stimulation indices (S.Is) (2.64 and 2.26, respectively), comparable to those recorded for TNF-α-matured DCs (3.09). Addition of mAb to MHC class II molecules reduced mean S.Is in all groups (0.87 for TNF-α; 0.93 for proTα; 0.98 for proTα(100–109)). These results suggest that following our *in vitro* culture protocol, peptide-reactive T cells are generated, which proliferate only in a HER-2(15_776_)-dependent MHC class II-restricted manner.

**Table 1:** **T cells stimulated with proTα- or proTα(100–109)-matured DCs proliferate in the presence of HER-2(15**_**776**_**)-pulsed DCs**

**DCs matured with**	**DCs pulsed with**	Mean counts per minute (cpm) ± SD*	Stimulation index (S.I.) ± SD*
TNF-α	-	13693 ± 1413	1
	HER-2(15_776_)	42314 ± 7139	3.09 ± 0.52
	tyr(15_448_)	16433 ± 1840	1.20 ± 0.13
	HER-2(15_776_) + anti-MHC class II	11914 ± 2033	0.87 ± 0.15
ProTα	-	13145 ± 1742	1
	HER-2(15_776_)	34702 ± 5143	2.64 ± 0.39
	tyr(15_448_)	17220 ± 2974	1.31 ± 0.23
	HER-2(15_776_) + anti-MHC class II	12225 ± 2603	0.93 ± 0.20
ProTα(100–109)	-	14577 ± 1041	1
	HER-2(15_776_)	32944 ± 6567	2.26 ± 0.45
	tyr(15_448_)	15306 ± 3608	1.05 ± 0.25
	HER-2(15_776_) + anti-MHC class II	14285 ± 2989	0.98 ± 0.20

* Mean cpm, S.I. ± SD from 2–5 donors.

### ProTα and proTα(100–109) induce the maturation of DCs *via* triggering TLR-4

**Figure 6:**
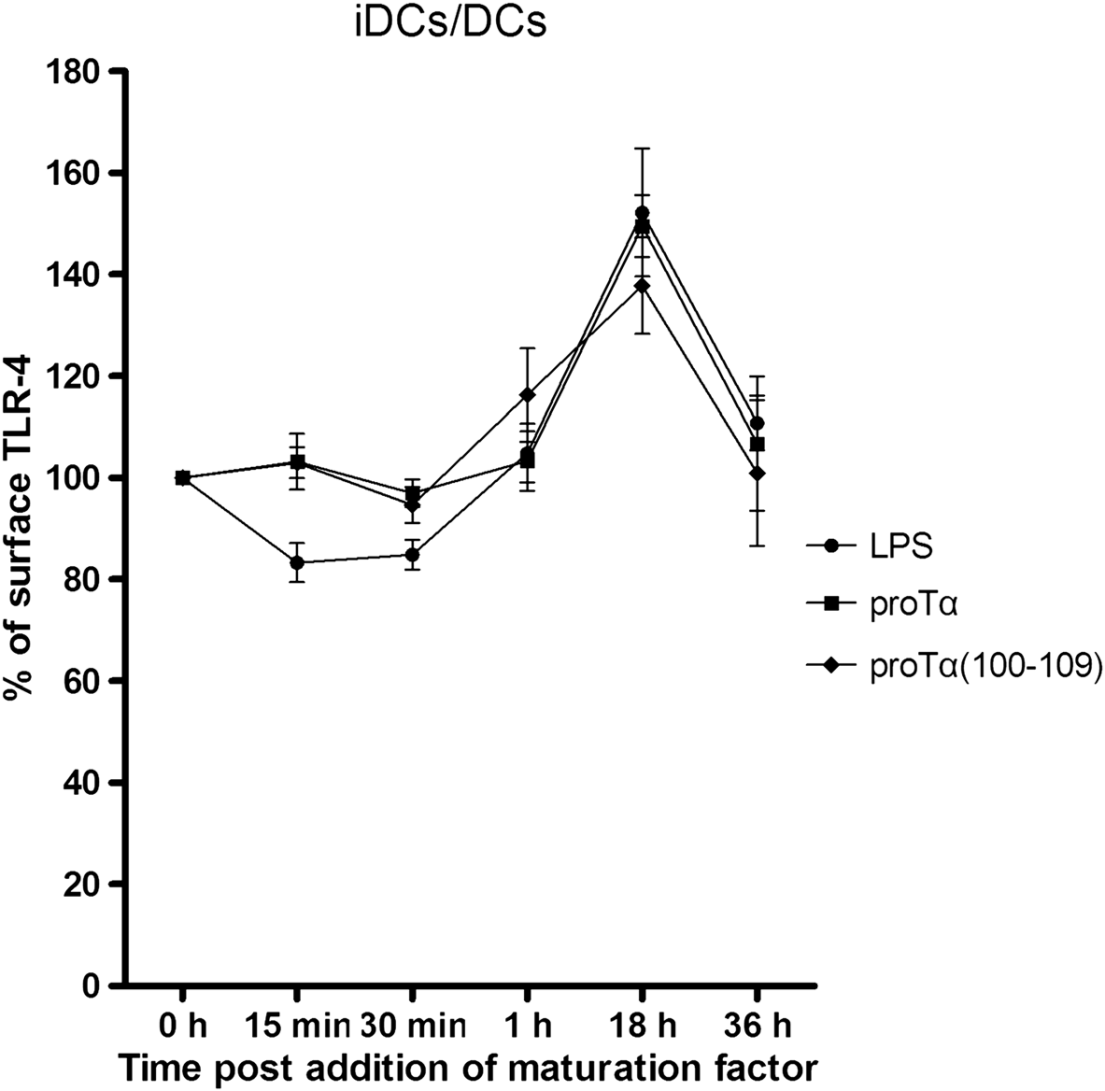
**Expression of TLR-4 on DCs matured with LPS, proTα or proαα(100–109).** iDCs differentiated from human monocytes (0 h) were matured with LPS, proαα and proαα(100–109) for 15 min, 30 min, 1 h, 18 h and 36 h, and analyzed for TLR-4 surface expression. Data show mean TLR-4 surface expression ± SD from 3 donors.

We have previously reported that stimulation of human monocytes with proTα upregulated IRAK-4, a protein kinase involved in TLR downstream signaling [19], whereas Mosoian *et al*. [20] showed that proTα ligates TLR-4 and signals through both TRIF- and MyD88-dependent pathways. To determine whether TLR-4 is triggered by our peptides, we studied the kinetics of TLR-4 surface expression on proTα and proTα(100–109)-stimulated DCs. Immature DCs (iDCs) and DCs matured with LPS (a known TLR-4 ligand; [30]), proTα or proTα(100–109) for 15 min, 30 min, 1 h, 18 h and 36 h were analyzed by flow cytometry. The percentage of surface TLR-4 expression over time is presented in Figure 6. Maturation of DCs with LPS led to an early (15 and 30 min) decrease of TLR-4 expression (by ~15%) due to internalization [31], and a subsequent increase from 1 to 18 h [32]. At 36 h post-LPS addition, TLR-4 expression was lower and comparable to that of iDCs (0 h). ProTα and proTα(100–109), marginally downregulated TLR-4 expression at 30 min and, similarly to LPS, transiently increased it from 1 to 18 h. As with LPS-matured DCs, basal levels of TLR-4 expression were detected at 36 h post-maturation. The similar kinetics of TLR-4 expression in LPS-, proTα- and proTα(100–109)-matured DCs is consistent with the notion that the two peptides interact with TLR-4.

**Figure 7:**
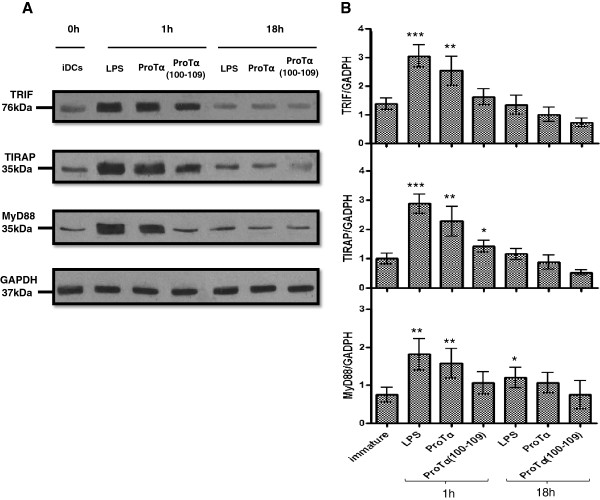
**Increased TLR-4-associated adaptor molecule expression in human DCs matured with proTα or proTα(100–109). (A)** Representative immunoblots of TRIF, TIRAP and MyD88 expression in cell extracts of iDCs and DCs matured with LPS, proTα or proTα(100–109) for 1 h and 18 h. GAPDH was used to verify equal loading. **(B)** Densitometry analysis of the adaptor proteins TRIF, TIRAP and MyD88, expressed as relative (%) expression *versus* GAPDH. Mean values of 4 independent experiments ± SD are shown. * *p* < 0.05; ** *p* < 0.01; *** *p* < 0.001.

To extend these findings, we next investigated the intracellular expression levels of three adaptor molecules that participate in signaling pathways downstream of TLR-4, namely TRIF, an adaptor molecule common to TLR-3 and -4 signaling; TIRAP, a signaling adaptor common to TLR-2, and -4; and MyD88, a molecule upregulated upon ligation of all TLRs except TLR-3 [33]. We specifically selected these three adaptors because this constellation is unique to TLR-4 activation. Total cell extracts from iDCs and DCs matured with LPS, proTα or proTα(100–109) for 1 h and 18 h were immunoblotted (Figure 7A). Upon densitometric quantification of each protein band detected, expression relative to GAPDH was calculated. As shown in Figure 7B, addition of LPS led to a significant ~2-3 fold increase of the expression of all three adaptors within 1 h (3.05 for TRIF, 2.88 for TIRAP and 1.81 for MyD88) relative to iDCs (1.38 for TRIF, 1.00 for TIRAP and 0.74 for MyD88). At 18 h post-addition of LPS, expression of all adaptors was decreased and again comparable to iDCs (1.35 for TRIF, 1.16 for TIRAP and 1.20 for MyD88). A similar trend of increased expression was also observed 1 h after addition of proTα (2.537 for TRIF, 2.28 for TIRAP and 1.577 for MyD88) or proTα(100–109) (1.62 for TRIF, 1.423 for TIRAP, 1.06 for MyD88), although in the latter case, the detected protein levels were lower. As with LPS, 18 h after proTα or proTα(100–109) DC-stimulation, the expression of TRIF, TIRAP and MyD88 was reduced and was similar to iDCs. These data, in conjunction with the cytokine profile shown in Figure 2, suggest that LPS, proTα, and possibly also proTα(100–109), activate DCs at least partly through one common TLR-4-dependent intracellular signaling pathway.

## Discussion

We have previously shown that human monocyte-derived iDCs activated *in vitro* with proTα or its immunoreactive decapeptide, proTα(100–109), acquire a mature DC phenotype [23]. Here, we show that DC maturation induced by proTα or proTα(100–109) promotes the secretion of IL-12, rather than IL-10, from these cells. Thus, both proTα- and proTα(100–109)-matured DCs possess immunostimulatory properties appropriate for the efficient activation of T cells, through their enhanced antigen-presenting capacity (HLA-DR; signal 1), the increased expression of co-stimulatory molecules (CD80/CD86; signal 2) and the secretion of inflammatory mediators (IL-12), recently proposed to act as signal 3 for optimizing effector T cell functions [34,35].

We assessed whether these *ex vivo* generated DCs can present tumor-associated immunogenic peptides to autologous T cells, along with the appropriate signals for their activation. We pulsed DCs with one MHC class I- and one class II-restricted immunodominant epitope from the oncoprotein HER-2/neu, HER-2(9_369_) and HER-2/neu(15_776_), respectively [36,37]. Our results show that proTα- or proTα(100–109)-matured HER-2/neu peptide-pulsed DCs favor the generation of T_H_1-type immune responses *in vitro*, by polarizing CD4+ T cells to produce pro-inflammatory cytokines. This cytokine milieu, characterized by high levels of IFN-γ and IL-2, results in the generation of strong CD8+ T cell responses [26,38], as we also observed. Indeed, CD8+ effectors recovered from the same stimulation cultures exhibited a pro-inflammatory cytokine profile similar to the CD4+ T cells (Additional file 1: Tables S1A and B) and enhanced HER-2(9_369_)-specific MHC class I-restricted cytotoxicity. Of interest, a high percentage of the peptide-specific CD8+ T cells generated in our stimulation cultures were polyfunctional, a quality reportedly associated with superior T cell performance [28,29,39]. These findings, in conjunction with the observed enhancement of HER-2(15_776_)-specific T cell proliferation, suggest that in the presence of tumor antigenic peptides, proTα- and proTα(100–109)-matured DCs efficiently promote the expansion of peptide-specific T cells.

Different DC-stimulating agents, including TLR ligands, have long been and still are being explored to optimize the immunostimulatory properties of DCs [10,11,40,41]. Although it was initially proposed that TLRs recognized only PAMPs, accumulating evidence to date suggests that TLRs also bind and respond to endogenous ligands released during tissue injury and inflammation, termed DAMPs or “alarmins” [42]. Most prominent among the alarmins are HMGB1, members of the HSP family and granulysin [43], all of which mature and activate DCs *in vitro* and bias immune responses towards a T_H_1-type, when used as vaccine adjuvants *in vivo*[44-48]. We and others have previously shown that proTα promotes antigen-specific adaptive immune responses [20,49-52] and based on the data presented herein, we now identify proTα as an alarmin. Moreover, in line with data on immunoreactive peptide-fragments derived from either HMGB1 (Hp91; [53]) or HSP70 (HSP70_359-610_; [46]), we show that the immunologically active site of proTα, the decapeptide proTα(100–109) [23], also favors T_H_1-polarization and induces HER-2/neu peptide-specific immune responses.

To suggest a possible molecular mechanism underlying the effect of proTα and proTα(100–109), and considering recent data from ourselves and others [19,20], we investigated whether TLR-4 expressed on human mature DCs is triggered by proTα or proTα(100–109). Our results show that proTα- or proTα(100–109)-induced DC maturation was associated with modulation of TLR-4 surface expression. Moreover, the expression of three TLR-4-associated intracellular adaptors, TRIF, TIRAP and MyD88, was promptly (at 1 h post-stimulation) increased in proTα- or proTα(100–109)-matured DCs, providing indirect evidence that the adjuvant activity of proTα and proTα(100–109) most likely involves TLR-4. Our data are in agreement with those of Mosoian *et al*. [20], showing that in murine macrophages proTα signals through the MyD88- and the TRIF-dependent pathways inducing TNF-α and type I IFN production, respectively. TLR ligation is a common mechanism of action, shared by different DAMPs. TLR-2 and -4 are involved in HMGB1 signaling *in vitro*[54-56], and several HSPs, including HSP22, HSP60, HSP70 and HSP90 also act as TLR-4 agonists [17,57-59]. Our results add to these observations, suggesting that both proTα and its shorter immunoactive decapeptide likely signal through TLR-4. The ambiguities raised as to whether proTα and proTα(100–109) share a common mechanism of action on DCs with LPS, could be attributed to: (1) inadequate internalization of TLR-4 by monocyte-derived human DCs, which reportedly are CD14^low^ (Figure 1, Additional file 2: Figure S1; [60,61]). Indeed, stimulation of CD14^high^ human monocytes and monocyte-derived human macrophages (Additional file 3: Figure S2) with proTα or proTα(100–109), induced the rapid CD14-dependent endocytosis of TLR-4, with kinetics similar to the response to LPS (Additional file 2: Figure S1); (2) differential requirements for TLR-4-mediated signaling depending on the cell population (eg. monocytes, macrophages *versus* DCs; [62]) and/or cell origin (eg. mouse *versus* human; [63]); and (3) the involvement of other TLRs (eg. TLR-2) and/or PRRs in proTα- and proTα(100–109)-induced DC signaling. In support of the latter, a similar phenomenon has been described for HMGB1; the intact protein signals through TLR-2 and -4 [53], and its immunostimulatory peptide Hp91 acts *via* TLR-3 or even other receptors [45].

## Conclusion

Taken altogether, we show herein that proTα and proTα(100–109) optimize immunogenic peptide-pulsed DC functionalities *in vitro*, possibly by TLR-4 triggering. *Ex vivo* education of DCs by proTα or proTα(100–109) results in their polarization to type-1 DCs, with increased capacity to stimulate tumor peptide-specific T cell responses and to render cytotoxic T cells polyfunctional. If this holds true also *in vivo*, then these molecules could be promising components of DC-based anti-cancer vaccines.

## Methods

### Peptide synthesis

ProTα(100–109), and the tumor antigen epitopes HER-2(9_369_), tyr(9_369_) (HLA-A2-restricted) [64], HER-2(15_776_) and tyr(15_448_) (HLA-DR4-restricted) [36,64] were synthesized by the Fmoc (9-fluorenylmethoxycarbonyl)/tBu chemistry utilizing a multiple peptide synthesizer Syro II (MultiSynTech, Witten, Germany). Crude peptides were purified by HPLC on a reverse phase C18 Nucleosil 100-5C column (HPLC Technologies, UK) to a purity of >95%, using a linear gradient of 5.8% acetonitrile in 0.05% trifluoroacetic acid for 45 min. All peptides were characterized by matrix-assisted laser desorption ionization-time of flight mass spectrometry and results were in all cases in agreement with the calculated masses. Human recombinant proTα was purchased from Alexis Biochemicals, CA, USA and passed through an Endotoxin removal column (Pierce Biotechnology). Prior to their use, all peptides and proTα were tested for endotoxin levels using the LAL chromogenic Endotoxin Quantitation kit (Pierce Biotechnology, IL, USA) according to the manufacturer’s instructions. They were endotoxin-free.

### Cell lines and PBMC isolation

Human T2 cells (HLA-A*0201) were cultured in RPMI 1640, supplemented with 10% heat-inactivated fetal bovine serum (FBS), 2 mM L-glutamine, 10 mM Hepes, 5 μg/mL Gentamycin, 10 U/mL Penicillin and 10 U/mL Streptomycin (all from Lonza, Cologne, Germany), at 37°C, in a humidified 5% CO_2_ incubator.

Buffy coats were collected from HLA-A2+ and DR4+ healthy blood donors. Prior to blood draw, individuals gave their informed consent according to the regulations approved by the 2nd Peripheral Blood Transfusion Unit and Haemophilia Centre, ‘Laikon’ General Hospital Institutional Review Board, Athens, Greece. PBMCs were isolated by centrifugation over Ficoll-Histopaque (Lonza) density gradient, resuspended in X-VIVO 15 (Lonza) or cryopreserved in FBS-10% DMSO (Sigma-Aldrich Chemical Co., St Louis, MO, USA) for later use.

### DC maturation and T cell stimulation

Highly enriched monocytes (>80% CD14+) were obtained from PBMCs by plastic adherence for 2 h at 37°C [65]. Non-adherent cells were removed and cryopreserved. Monocytes were cultured for 5 days in X-VIVO 15 supplemented with 800 IU/mL recombinant human granulocyte macrophage colony-stimulating factor (GM-CSF) and 500 IU/mL recombinant human IL-4 (both from R&D Systems GmbH, Wiesbaden-Nordenstadt, Germany). On day 5, iDCs were treated with LPS (0.5 μg/mL; Sigma-Aldrich), TNF-α (10 ng/mL; R&D Systems), proTα (160 ng/mL) or proαα(100–109) (25 ng/mL) for 1–48 h, concentrations already reported to induce DC maturation [23]. Mature DCs were recovered at various time points for phenotypic and TLR-4 analysis by flow cytometry and immunoblotting, or were used to stimulate autologous T cells. Supernatants from 48 h matured DCs were also collected and the concentrations of TNF-α, IL-10 and IL-12 were quantified using commercially available ELISA kits (all from Life Technologies Corporation, Carlsbad, USA), according to manufacturer’s instructions. For TLR-4 neutralization experiments, iDCs were pre-incubated in the presence of anti-TLR-4 (a-TLR-4) neutralizing monoclonal antibody (mAb; clone W7C11) or an irrelevant mouse IgG1 mAb (both from InvivoGen, San Diego, USA) at a final concentration of 10 μg/mL for 1 h and further stimulated with LPS, proTα or proTα(100–109) for 48 h. TNF-α, IL-10 and IL-12 were determined in culture supernatants.

For T cell stimulation, 48 h matured DCs (1×10^6^/mL) were pulsed with 50 μg/mL HER-2(9_369_) and HER-2(15_776_) for 6 h at 37°C, in a humidified 5% CO_2_ incubator in X-VIVO 15. DCs were washed twice, resuspended in X-VIVO 15 and added to autologous lymphocytes (non-adherent fraction) at a DC:lymphocyte ratio of 1:10. T cells were stimulated thrice at weekly intervals and on days 3 and 5 after each stimulation, 40 IU/mL IL-2 (Proleukin; Novartis Pharmaceuticals Ltd, UK) were added to the cultures. At the third stimulation, Golgi-Plug (1 μL/mL; Becton-Dickinson (BD) Biosciences, Erembodegem, Belgium) was added in the cultures, and 12 h later, T cells were harvested and analyzed for cytokine production by flow cytometry.

### Flow cytometry analysis

For DC phenotype analysis, iDCs and mature DCs were stained for the surface molecules HLA-DR, CD80, CD83, CD86, CD11b, CD40 and CD14. Triple staining was performed using appropriate combinations of FITC-, PE- or PE-Cy5-labelled mouse anti-human IgG1 and IgG2 mAbs (BD Biosciences) at saturating concentrations for 30 min on ice. DCs were also stained with irrelevant anti-human IgG1 and IgG2 mAbs (BD Biosciences), as isotype controls. Samples were measured using a FACSCalibur flow cytometer (BD Biosciences) and data were analyzed using CellQuest software. MFI was evaluated for each marker.

For TLR-4 expression, iDCs and DCs matured with LPS, proTα or proTα(100–109) for 15 min, 30 min, 1 h, 18 h and 36 h were harvested and treated with human immunoglobulin (GAMUNEX; Bayer, Leverkusen, Germany) and ethidium monoazide (EMA; Invitrogen, Karlsruhe, Germany) to block Fc receptors and label nonviable cells, respectively. DCs were then stained with TLR-4/Brilliant Violet 421, CD11c/PE-Cy7 (both from BioLegend, San Diego, CA) and Lineage 1 cocktail/FITC (BD Biosciences) mAbs and measured immediately using LSR II or FACSCanto II and FACSDiva software (BD Biosciences). Data were analyzed using FlowJo software (TreeStar, Ashland, OR). Duplicates were excluded using the forward-scatter area *versus* forward-scatter height plot, TLR-4+ cells were gated within viable DCs (EMA-negative (−), CD11c + and Lineage 1-) and their MFI was determined. For TLR-4 neutralization experiments, a-TLR-4-treated iDCs were stimulated as above and stained with CD14/FITC (BioLegend) and TLR-4/Brilliant Violet 421 or PE (BioLegend) mAbs at saturating concentrations for 30 min on ice. DCs were also stained with irrelevant anti-human IgG2 mAbs (BD Biosciences), as isotype controls. Samples were measured using a FACSCanto II and data were analyzed using FACSDiva software.

For cytokine production analysis, T cells were harvested and treated with GAMUNEX and EMA. They were then stained with the following mAbs: CD3/eFluor 605, IL-10/PE, and IL-17/PerCP-Cy5.5 (eBioscience, San Diego, CA); CD-4/PerCP, CD-8/APC-H7, IL-4/APC, IFN-γ/PE-Cy7 and CD107a/FITC (BD Biosciences); IL-2/Alexa700 and TNF-α/Brilliant Violet 421 (BioLegend). Samples were analysed immediately using an LSR II and FACSDiva software and data were processed using FlowJo software. Duplicates were excluded using the forward-scatter area *versus* forward-scatter height plot, and CD4+ and CD8+ cells were gated within viable CD3+ lymphocytes and analyzed separately for cytokine production. The percentage of cells producing each cytokine on gated T cells was determined.

### Cytotoxicity assay

The cytotoxic activity of thrice stimulated T cells was determined by standard ^51^Cr- release assay. T2 cells were incubated for 2 h at 37°C with 10 μg/mL HER-2(9_369_) or tyr(9_369_), washed and labeled with sodium chromate, as previously described [21]. Non-loaded T2 were similarly labeled for controls. Effectors (1×10^6^/mL in X-VIVO 15; 100 μL/well) were seeded in 96-well U-bottom plates (Greiner Bio-one, Kirchheim, Germany) and T2 targets were added (5×10^4^/mL; 100 μL/well), at an effector:target (E:T) ratio of 10:1. Where indicated, mAb to MHC class I molecules (W6/32, kindly donated by Prof. S. Stevanovic, University of Tübingen) was added to the cultures at a final concentration of 5 μg/mL for the entire incubation period [66]. After 18 h of coincubation at 37°C, 5% CO_2_, 100 μL of supernatant were removed from each well and isotope (counts per minute (cpm)) was counted in a γ-counter (1275 Mini-gamma LKB Wallac, Turku, Finland). To determine maximal and spontaneous isotope release, targets were incubated with 3 N HCl and in plain medium, respectively. All cultures were set in triplicate. Percentage of specific cytotoxicity was calculated according to the formula: [(cpm experimental-cpm spontaneous)/(cpm maximal-cpm spontaneous)] ×100.

### Proliferation assay

Stimulated T cells were seeded in 96-well U-bottom plates (1 × 10^6^/mL; 100 μL). Autologous matured DCs pulsed with 50 μg/mL HER-2(15_776_) or tyr(15_448_) for 6 h, were added (1 × 10^5^/mL; 100 μL/well) and cocultured for 5 days. T cells incubated with unpulsed matured DCs or in the presence of IL-2 (500 IU/mL) were used as controls. Where indicated, mAb to MHC class II molecules (L243, kindly donated by Prof. S. Stevanovic) was added to the cultures at a concentration of 5 μg/mL for the entire culture period [66]. For the last 18 h of culture, 1 μCi ^3^H-thymidine (Amersham Pharmacia Biotech, Amersham, Bucks, UK) was added per well and cells were harvested in a semi-automatic cell harvester (Skatron Inc., Tranby, Norway). The amount of incorporated radioactivity, proportional to DNA synthesis, was measured in a liquid scintillation counter (Wallac, Turku, Finland) and expressed as cpm. The S.I. of each experimental group was calculated using the formula: (average cpm of sample in the presence of peptide-pulsed DCs)/(average cpm of sample in the presence of unpulsed DCs).

### Immunoblotting

Total cell extracts from 4–5×10^5^ iDCs and DCs matured with LPS, proTα or proTα(100–109) were extracted as described [67]. Briefly, cells were lysed in NP-40 lysis buffer (1% NP-40, 150 mM NaCl, 50 mM Tris pH 8.0) containing protease inhibitors (Protease Inhibitor Cocktail, Sigma-Aldrich) and lysates were cleared by centrifugation for 10 min at 19,000 g (4°C). The protein content of extracts was determined by the Bradford assay, samples were mixed with reducing Laemmli buffer and equal protein amounts (15–25 μg) were separated by sodium dodecyl sulfate–polyacrylamide gel electrophoresis using 12% (w/v) polyacrylamide gels. Separated proteins were blotted on nitrocellulose membranes and probed with primary antibodies (goat anti-human αRIF/Novus Biologicals, Ltd, Cambridge, UK; rabbit anti-human MyD88 and rabbit anti-human TIRAP/eBioscience; rabbit anti-human GAPDH/Santa Cruz Biotechnology Inc, Santa Cruz, CA, USA) and horseradish peroxidase (HRP)-conjugated secondary antibodies (anti-rabbit-IgG and anti-goat-IgG/Santa Cruz Biotechnology). Immunoblots were developed using an enhanced chemiluminescence reagent kit (Santa Cruz Biotechnology) and quantified by scanning densitometry (Gel Analyzer v.1.0, Biosure, Athens, Greece).

### Statistical analysis

Data were analyzed by the Student’s t-test and statistical significance was presumed at significance level of 5% (*p* < 0.05).

## Abbreviations

HMGB1: High mobility group box 1; HSP: Heat shock protein; MFI: Mean fluorescence intensity; proTα: Prothymosin alpha; TLR: Toll-like receptor.

## Competing interests

The authors declare that they have no competing interests.

## Authors’ contributions

KI: performed the experiments, analyzed data, carried out statistical analyses and wrote the manuscript. ED: designed, analysed and interpreted flow cytometry data and helped to write the manuscript. ET: participated in immunoblotting data acquisition and analyses. PS: performed sample collection and helped to draft the manuscript. HK: carried out peptide synthesis and purification and helped to draft the manuscript. WV: helped in HLA-typing and to draft the manuscript. IPT: participated in the design of the study and reviewed the manuscript. GP: participated in the design and coordination of the study, helped to draft, reviewed and edited the manuscript. OET: conceived, designed and coordinated the study, drafted and reviewed the manuscript. All authors read and approved the final manuscript.

## Supplementary Material

Additional file 1: Table S1 Range of % cytokine positive CD4+ and CD8+ T cells. (A) Intracellular production of IFN-γ, TNF-α, IL-2, IL-4, IL-10 and IL-17 in, and expression of CD107 on CD4+ and CD8+ T cells stimulated with DCs matured with TNF-α, proTα or proTα(100–109), in the absence (−) or presence (+) of the HER-2/neu peptides. IL-5+ and IL-13+ CD8+ T cells are additionally shown. Numbers indicate percentages of positive cells. Shown is the range detected from 3–5 different donors tested. (B) Ratios of IFN-γ/IL-5 and IFN-γ/IL-13 in CD8+ T cells. Shown is the range from 3 different donors tested. Click here for file

Additional file 2: Figure S1 Kinetics of CD14 and TLR-4 surface expression on monocytes, macrophages and iDCs/DCs upon stimulation with LPS, proTα or proTα(100–109). Monocytes, macrophages and iDCs (0 h) were stimulated with LPS (A), proTα (B), or proTα(100–109) (C) for 15 min, 30 min, 1 h and 18 h and assessed for the surface expression of CD14 and TLR-4 using flow cytometry. MFI values in the presence of neutralizing anti-TLR-4 Ab (+ a-TLR-4) are shown below each histogram. Histograms are from one representative donor of 3 tested. Using the loss of cell surface expression as a readout for TLR-4 and CD14 endocytosis from 0–36 h [31], data from all three donors are shown as mean values ± SDs for TLR-4 (D, E, F) and CD14 (G, H, I). Click here for file

Additional file 3: Figure S2 CD14, TLR-4 and CD206 expression on monocytes, monocyte-derived macrophages and monocyte-derived iDCs. Macrophages were generated from human monocytes upon incubation with 100 ng/mL GM-CSF for 5 days. Human monocytes were isolated and iDCs were generated as described in Methods. Monocytes, macrophages and iDCs were assessed for the surface expression of CD14, TLR-4 and CD206 (as a specific marker for macrophages and DCs), using flow cytometry. Histograms are from one representative donor of 3 tested and numbers indicate MFIs. Click here for file

## References

[B1] Chianese-Bullock KA Irvin WP Jr Petroni GR Murphy C Smolkin M Olson WC Coleman E Boerner SA Nail CJ Neese PY Yuan A Hogan KT Slingluff CL Jr A multipeptide vaccine is safe and elicits T-cell responses in participants with advanced stage ovarian cancer J Immunother 2008 31 420 430 10.1097/CJI.0b013e31816dad10 18391753

[B2] Barve M Bender J Senzer N Cunningham C Greco FA McCune D Steis R Khong H Richards D Stephenson J Ganesa P Nemunaitis J Ishioka G Pappen B Nemunaitis M Morse M Mills B Maples PB Sherman J Nemunaitis JJ Induction of immune responses and clinical efficacy in a phase II trial of IDM-2101, a 10-epitope cytotoxic T-lymphocyte vaccine, in metastatic non-small-cell lung cancer J Clin Oncol 2008 26 4418 4425 10.1200/JCO.2008.16.6462 18802154

[B3] Walter S Weinschenk T Stenzl A Zdrojowy R Pluzanska A Szczylik C Staehler M Brugger W Dietrich PY Mendrzyk R Hilf N Schoor O Fritsche J Mahr A Maurer D Vass V Trautwein C Lewandrowski P Flohr C Pohla H Stanczak JJ Bronte V Mandruzzato S Biedermann T Pawelec G Derhovanessian E Yamagishi H Miki T Hongo F Takaha N Hirakawa K Tanaka H Stevanovic S Frisch J Mayer-Mokler A Kirner A Rammensee HG Reinhardt C Singh-Jasuja H Multipeptide immune response to cancer vaccine IMA901 after single-dose cyclophosphamide associates with longer patient survival Nat Med 2012 18 1254 1261 10.1038/nm.2883 22842478

[B4] Coffman RL Sher A Seder RA Vaccine adjuvants: putting innate immunity to work Immunity 2010 33 492 503 10.1016/j.immuni.2010.10.002 21029960 PMC3420356

[B5] Jähnisch H Füssel S Kiessling A Wehner R Zastrow S Bachmann M Rieber EP Wirth MP Schmitz M Dendritic cell-based immunotherapy for prostate cancer Clin Dev Immunol 2010 2010 517493 21076523 10.1155/2010/517493 PMC2975068

[B6] Palucka AK Ueno H Fay J Banchereau J Dendritic cells: a critical player in cancer therapy? J Immunother 2008 31 793 805 10.1097/CJI.0b013e31818403bc 18833008 PMC2716088

[B7] Palucka K Ueno H Fay J Banchereau J Dendritic cells and immunity against cancer J Intern Med 2011 269 64 73 10.1111/j.1365-2796.2010.02317.x 21158979 PMC3023888

[B8] Schreibelt G Benitez-Ribas D Schuurhuis D Lambeck AJ van Hout-Kuijer M Schaft N Punt CJ Figdor CG Adema GJ de Vries IJ Commonly used prophylactic vaccines as an alternative for synthetically produced TLR ligands to mature monocyte-derived dendritic cells Blood 2010 116 564 574 10.1182/blood-2009-11-251884 20424184

[B9] Pinzon-Charry A Maxwell T López JA Dendritic cell dysfunction in cancer: a mechanism for immunosuppression Immunol Cell Biol 2005 83 451 461 10.1111/j.1440-1711.2005.01371.x 16174093

[B10] Schilling B Harasymczuk M Schuler P Egan J Ferrone S Whiteside TL IRX-2, a novel immunotherapeutic, enhances functions of human dendritic cells PLoS One 2013 8 e47234 10.1371/journal.pone.0047234 23408925 PMC3567103

[B11] Napoletano C Zizzari IG Rughetti A Rahimi H Irimura T Clausen H Wandall HH Belleudi F Bellati F Pierelli L Frati L Nuti M Targeting of macrophage galactose-type C-type lectin (MGL) induces DC signaling and activation Eur J Immunol 2012 42 936 945 10.1002/eji.201142086 22531918

[B12] Vacchelli E Galluzzi L Eggermont A Fridman WH Galon J Sautès-Fridman C Tartour E Zitvogel L Kroemer G Trial watch: FDA-approved Toll-like receptor agonists for cancer therapy Oncoimmunology 2012 1 894 907 10.4161/onci.20931 23162757 PMC3489745

[B13] Karbach J Gnjatic S Bender A Neumann A Weidmann E Yuan J Ferrara CA Hoffmann E Old LJ Altorki NK Jäger E Tumor-reactive CD8+ T-cell responses after vaccination with NY-ESO-1 peptide, CpG 7909 and Montanide ISA-51: association with survival Int J Cancer 2010 126 909 918 19728336 10.1002/ijc.24850

[B14] Wieckowski E Chatta GS Mailliard RM Gooding W Palucka K Banchereau J Kalinski P Type-1 polarized dendritic cells loaded with apoptotic prostate cancer cells are potent inducers of CD8(+) T cells against prostate cancer cells and defined prostate cancer-specific epitopes Prostate 2011 71 125 133 10.1002/pros.21228 20717900 PMC2989344

[B15] Feyerabend S Stevanovic S Gouttefangeas C Wernet D Hennenlotter J Bedke J Dietz K Pascolo S Kuczyk M Rammensee HG Stenzl A Novel multi-peptide vaccination in Hla-A2+ hormone sensitive patients with biochemical relapse of prostate cancer Prostate 2009 69 917 927 10.1002/pros.20941 19267352

[B16] Cluff CW Monophosphoryl lipid A (MPL) as an adjuvant for anti-cancer vaccines: clinical results Adv Exp Med Biol 2010 667 111 123 20665204 10.1007/978-1-4419-1603-7_10

[B17] Butler GS Overall CM Proteomic identification of multitasking proteins in unexpected locations complicates drug targeting Nat Rev Drug Discov 2009 8 935 948 10.1038/nrd2945 19949400

[B18] Ioannou K Samara P Livaniou E Derhovanessian E Tsitsilonis OE Prothymosin alpha: a ubiquitous polypeptide with potential use in cancer diagnosis and therapy Cancer Immunol Immunother 2012 61 599 614 10.1007/s00262-012-1222-8 22366887 PMC11029552

[B19] Skopeliti M Kratzer U Altenberend F Panayotou G Kalbacher H Stevanovic S Voelter W Tsitsilonis OE Proteomic exploitation on prothymosin alpha-induced mononuclear cell activation Proteomics 2007 7 1814 1824 10.1002/pmic.200600870 17474146

[B20] Mosoian A Teixeira A Burns CS Sander LE Gusella GL He C Blander JM Klotman P Klotman ME Prothymosin-alpha inhibits HIV-1 via Toll-like receptor 4-mediated type I interferon induction Proc Natl Acad Sci USA 2010 107 10178 10183 10.1073/pnas.0914870107 20479248 PMC2890444

[B21] Skopeliti M Voutsas IF Klimentzou P Tsiatas ML Beck A Bamias A Moraki M Livaniou E Neagu M Voelter W Tsitsilonis OE The immunologically active site of prothymosin alpha is located at the carboxy-terminus of the polypeptide. Evaluation of its in vitro effects in cancer patients Cancer Immunol Immunother 2006 55 1247 1257 10.1007/s00262-005-0108-4 16453152 PMC11030181

[B22] Baxevanis CN Thanos D Reclos GJ Anastasopoulos E Tsokos GC Papamatheakis J Papamichail M Prothymosin alpha enhances human and murine MHC class II surface antigen expression and messenger RNA accumulation J Immunol 1992 148 1979 1984 1545115

[B23] Skopeliti M Iconomidou VA Derhovanessian E Pawelec G Voelter W Kalbacher H Hamodrakas SJ Tsitsilonis OE Prothymosin alpha immunoactive carboxyl-terminal peptide TKKQKTDEDD stimulates lymphocyte reactions, induces dendritic cell maturation and adopts a beta-sheet conformation in a sequence-specific manner Mol Immunol 2009 46 784 792 10.1016/j.molimm.2008.09.014 18976813

[B24] Kapsenberg ML Dendritic-cell control of pathogen-driven T-cell polarization Nat Rev Immunol 2003 3 984 993 10.1038/nri1246 14647480

[B25] Hovden AO Karlsen M Jonsson R Appel S The bacterial preparation OK432 induces IL-12p70 secretion in human dendritic cells in a TLR3 dependent manner PLoS One 2012 7 e31217 10.1371/journal.pone.0031217 22363584 PMC3283639

[B26] Bevan MJ Helping the CD8(+) T-cell response Nat Rev Immunol 2004 4 595 602 10.1038/nri1413 15286726

[B27] Rubio V Stuge TB Singh N Betts MR Weber JS Roederer M Lee PP Ex vivo identification, isolation and analysis of tumor-cytolytic T cells Nat Med 2003 9 1377 1382 10.1038/nm942 14528297

[B28] Darrah PA Patel DT De Luca PM Lindsay RW Davey DF Flynn BJ Hoff ST Andersen P Reed SG Morris SL Roederer M Seder RA Multifunctional TH1 cells define a correlate of vaccine-mediated protection against Leishmania major Nat Med 2007 13 843 850 10.1038/nm1592 17558415

[B29] Precopio ML Betts MR Parrino J Price DA Gostick E Ambrozak DR Asher TE Douek DC Harari A Pantaleo G Bailer R Graham BS Roederer M Koup RA Immunization with vaccinia virus induces polyfunctional and phenotypically distinctive CD8(+) T cell responses J Exp Med 2007 204 1405 1416 10.1084/jem.20062363 17535971 PMC2118607

[B30] Yamamoto M Akira S Lipid A receptor TLR4-mediated signaling pathways Adv Exp Med Biol 2010 667 59 68 20665200 10.1007/978-1-4419-1603-7_6

[B31] Zanoni I Ostuni R Marek LR Barresi S Barbalat R Barton GM Granucci F Kagan JC CD14 controls the LPS-induced endocytosis of Toll-like receptor 4 Cell 2011 147 868 880 10.1016/j.cell.2011.09.051 22078883 PMC3217211

[B32] Nagata A Takezako N Tamemoto H Ohto-Ozaki H Ohta S Tominaga S Yanagisawa K Soluble ST2 protein inhibits LPS stimulation on monocyte-derived dendritic cells Cell Mol Immunol 2012 9 399 409 10.1038/cmi.2012.29 22922442 PMC4002335

[B33] Takeda K Akira S TLR signaling pathways Semin Immunol 2004 16 3 9 10.1016/j.smim.2003.10.003 14751757

[B34] Navabi H Jasani B Reece A Clayton A Tabi Z Donninger C Mason M Adams M A clinical grade poly I:C-analogue (Ampligen) promotes optimal DC maturation and Th1-type T cell responses of healthy donors and cancer patients in vitro Vaccine 2009 27 107 115 10.1016/j.vaccine.2008.10.024 18977262

[B35] Kalinski P Edington H Zeh HJ Okada H Butterfield LH Kirkwood JM Bartlett DL Dendritic cells in cancer immunotherapy: vaccines or autologous transplants? Immunol Res 2011 50 235 247 10.1007/s12026-011-8224-z 21717071 PMC3695396

[B36] Salazar LG Fikes J Southwood S Ishioka G Knutson KL Gooley TA Schiffman K Disis ML Immunization of cancer patients with HER-2/neu-derived peptides demonstrating high-affinity binding to multiple class II alleles Clin Cancer Res 2003 9 5559 5565 14654536

[B37] Bernhard H Salazar L Schiffman K Smorlesi A Schmidt B Knutson KL Disis ML Vaccination against the HER-2/neu oncogenic protein Endocr Relat Cancer 2002 9 33 44 10.1677/erc.0.0090033 11914181

[B38] Green AM Difazio R Flynn JL IFN-γ from CD4 T cells is essential for host survival and enhances CD8 T cell function during Mycobacterium tuberculosis infection J Immunol 2013 190 270 277 10.4049/jimmunol.1200061 23233724 PMC3683563

[B39] Almeida JR Price DA Papagno L Arkoub ZA Sauce D Bornstein E Asher TE Samri A Schnuriger A Theodorou I Costagliola D Rouzioux C Agut H Marcelin AG Douek D Autran B Appay V Superior control of HIV-1 replication by CD8+ T cells is reflected by their avidity, polyfunctionality, and clonal turnover J Exp Med 2007 204 2473 2485 10.1084/jem.20070784 17893201 PMC2118466

[B40] Lichtenegger FS Mueller K Otte B Beck B Hiddemann W Schendel DJ Subklewe M CD86 and IL-12p70 are key players for T helper 1 polarization and natural killer cell activation by Toll-like receptor-induced dendritic cells PLoS One 2012 7 e44266 10.1371/journal.pone.0044266 22962607 PMC3433478

[B41] Jung ID Jeong SK Lee CM Noh KT Heo DR Shin YK Yun CH Koh WJ Akira S Whang J Kim HJ Park WS Shin SJ Park YM Enhanced efficacy of therapeutic cancer vaccines produced by co-treatment with Mycobacterium tuberculosis heparin-binding hemagglutinin, a novel TLR4 agonist Cancer Res 2011 71 2858 2870 10.1158/0008-5472.CAN-10-3487 21368092

[B42] Baxevanis CN Voutsas IF Tsitsilonis OE Toll-like receptor agonists: current status and future perspective on their utility as adjuvants in improving anticancer vaccination strategies Immunotherapy 2013 5 497 511 10.2217/imt.13.24 23638745

[B43] Bianchi ME DAMPs, PAMPs and alarmins: all we need to know about danger J Leukoc Biol 2007 81 1 5 17032697 10.1189/jlb.0306164

[B44] Messmer D Yang H Telusma G Knoll F Li J Messmer B Tracey KJ Chiorazzi N High mobility group box protein 1: an endogenous signal for dendritic cell maturation and Th1 polarization J Immunol 2004 173 307 313 15210788 10.4049/jimmunol.173.1.307

[B45] Saenz R Souza Cda S Huang CT Larsson M Esener S Messmer D HMGB1-derived peptide acts as adjuvant inducing immune responses to peptide and protein antigen Vaccine 2010 28 7556 7562 10.1016/j.vaccine.2010.08.054 20800114 PMC2963688

[B46] Wang Y Kelly CG Singh M McGowan EG Carrara AS Bergmeier LA Lehner T Stimulation of Th1-polarizing cytokines, C-C chemokines, maturation of dendritic cells, and adjuvant function by the peptide binding fragment of heat shock protein 70 J Immunol 2002 169 2422 2429 12193710 10.4049/jimmunol.169.5.2422

[B47] Wu Y Wan T Zhou X Wang B Yang F Li N Chen G Dai S Liu S Zhang M Cao X Hsp70-like protein 1 fusion protein enhances induction of carcinoembryonic antigen-specific CD8+ CTL response by dendritic cell vaccine Cancer Res 2005 65 4947 4954 10.1158/0008-5472.CAN-04-3912 15930317

[B48] Tewary P Yang D de la Rosa G Li Y Finn MW Krensky AM Clayberger C Oppenheim JJ Granulysin activates antigen-presenting cells through TLR4 and acts as an immune alarmin Blood 2010 116 3465 3474 10.1182/blood-2010-03-273953 20660289 PMC2981473

[B49] Cordero OJ Sarandeses C López-Rodríguez JL Nogueira M The presence and cytotoxicity of CD16+ CD2- subset from PBL and NK cells in long-term IL-2 cultures enhanced by Prothymosin-alpha Immunopharmacology 1995 29 215 223 10.1016/0162-3109(95)00057-Z 7542644

[B50] Eckert K Grünberg E Garbin F Maurer HR Preclinical studies with prothymosin alpha1 on mononuclear cells from tumor patients Int J Immunopharmacol 1997 19 493 500 10.1016/S0192-0561(97)00079-9 9637344

[B51] Baxevanis CN Gritzapis AD Spanakos G Tsitsilonis OE Papamichail M Induction of tumor-specific T lymphocyte responses in vivo by prothymosin alpha Cancer Immunol Immunother 1995 40 410 418 10.1007/BF01525392 7543022 PMC11037699

[B52] Voutsas IF Baxevanis CN Gritzapis AD Missitzis I Stathopoulos GP Archodakis G Banis C Voelter W Papamichail M Synergy between interleukin-2 and prothymosin alpha for the increased generation of cytotoxic T lymphocytes against autologous human carcinomas Cancer Immunol Immunother 2000 49 449 458 10.1007/s002620000132 11043852 PMC11037007

[B53] Telusma G Datta S Mihajlov I Ma W Li J Yang H Newman W Messmer BT Minev B Schmidt-Wolf IG Tracey KJ Chiorazzi N Messmer D Dendritic cell activating peptides induce distinct cytokine profiles Int Immunol 2006 18 1563 1573 10.1093/intimm/dxl089 16966494

[B54] Park JS Svetkauskaite D He Q Kim JY Strassheim D Ishizaka A Abraham E Involvement of toll-like receptors 2 and 4 in cellular activation by high mobility group box 1 protein J Biol Chem 2004 279 7370 7377 14660645 10.1074/jbc.M306793200

[B55] Park JS Gamboni-Robertson F He Q Svetkauskaite D Kim JY Strassheim D Sohn JW Yamada S Maruyama I Banerjee A Ishizaka A Abraham E High mobility group box 1 protein interacts with multiple Toll-like receptors Am J Physiol Cell Physiol 2006 290 C917 924 16267105 10.1152/ajpcell.00401.2005

[B56] Yu M Wang H Ding A Golenbock DT Latz E Czura CJ Fenton MJ Tracey KJ Yang H HMGB1 signals through toll-like receptor (TLR) 4 and TLR2 Shock 2006 26 174 179 10.1097/01.shk.0000225404.51320.82 16878026

[B57] Roelofs MF Boelens WC Joosten LA Abdollahi-Roodsaz S Geurts J Wunderink LU Schreurs BW van den Berg WB Radstake TR Identification of small heat shock protein B8 (HSP22) as a novel TLR4 ligand and potential involvement in the pathogenesis of rheumatoid arthritis J Immunol 2006 176 7021 7027 16709864 10.4049/jimmunol.176.11.7021

[B58] Vabulas RM Ahmad-Nejad P da Costa C Miethke T Kirschning CJ Häcker H Wagner HL Endocytosed HSP60s use toll-like receptor 2 (TLR2) and TLR4 to activate the toll/interleukin-1 receptor signaling pathway in innate immune cells J Biol Chem 2001 276 31332 31339 10.1074/jbc.M103217200 11402040

[B59] Vabulas RM Ahmad-Nejad P Ghose S Kirschning CJ Issels RD Wagner H HSP70 as endogenous stimulus of the Toll/interleukin-1 receptor signal pathway J Biol Chem 2002 277 15107 15112 10.1074/jbc.M111204200 11842086

[B60] Jiang XX Zhang Y Liu B Zhang SX Wu Y Yu XD Mao N Human mesenchymal stem cells inhibit differentiation and function of monocyte-derived dendritic cells Blood 2005 105 4120 4126 10.1182/blood-2004-02-0586 15692068

[B61] Jarnjak-Jankovic S Hammerstad H Saebøe-Larssen S Kvalheim G Gaudernack G A full scale comparative study of methods for generation of functional dendritic cells for use as cancer vaccines BMC Cancer 2007 7 119 10.1186/1471-2407-7-119 17608923 PMC1931601

[B62] Zanoni I Granucci F Differences in lipopolysaccharide-induced signaling between conventional dendritic cells and macrophages Immunobiology 2010 215 709 712 10.1016/j.imbio.2010.05.026 20579765

[B63] Bosisio D Polentarutti N Sironi M Bernasconi S Miyake K Webb GR Martin MU Mantovani A Muzio M Stimulation of toll-like receptor 4 expression in human mononuclear phagocytes by interferon-gamma: a molecular basis for priming and synergism with bacterial lipopolysaccharide Blood 2002 99 3427 3431 10.1182/blood.V99.9.3427 11964313

[B64] Robbins PF Nagorsen D, Marincola FM Tumor associated antigens Analyzing T cell responses 2005 Netherlands: Springer 9 42

[B65] Gavalas NG Tsiatas M Tsitsilonis O Politi E Ioannou K Ziogas AC Rodolakis A Vlahos G Thomakos N Haidopoulos D Terpos E Antsaklis A Dimopoulos MA Bamias A VEGF directly suppresses activation of T cells from ascites secondary to ovarian cancer via VEGF receptor type 2 Br J Cancer 2012 107 1869 1875 10.1038/bjc.2012.468 23169339 PMC3504940

[B66] Sun Y Stevanovic S Song M Schwantes A Kirkpatrick CJ Schadendorf D Cichutek K The kinase insert domain-containing receptor is an angiogenesis-associated antigen recognized by human cytotoxic T lymphocytes Blood 2006 107 1476 1483 10.1182/blood-2005-05-1912 16234362

[B67] Antonelou MH Kriebardis AG Stamoulis KE Trougakos IP Papassideri IS Apolipoprotein J/clusterin in human erythrocytes is involved in the molecular process of defected material disposal during vesiculation PLoS One 2011 6 e26033 10.1371/journal.pone.0026033 22016805 PMC3189238

