# Supplementary material for: Prothymosin α and a prothymosin α-derived peptide enhance TH1-type immune responses against defined HER-2/neu epitopes
Source: BMC Immunol. 2013 Sep 22;14:43. doi: 10.1186/1471-2172-14-43 (PMC3852324; doi:10.1186/1471-2172-14-43)
Supplement: Additional file 1: Table S1 — Range of % cytokine positive CD4+ and CD8+ T cells. (A) Intracellular production of IFN-γ, TNF-α, IL-2, IL-4, IL-10 and IL-17 in, and expression of CD107 on CD4+ and CD8+ T cells stimulated with DCs matured with TNF-α, proTα or proTα(100–109), in the absence (−) or presence (+) of the HER-2/neu peptides. IL-5+ and IL-13+ CD8+ T cells are additionally shown. Numbers indicate percentages of positive cells. Shown is the range detected from 3–5 different donors tested. (B) Ratios of IFN-γ/IL-5 and IFN-γ/IL-13 in CD8+ T cells. Shown is the range from 3 different donors tested. [file 1471-2172-14-43-S1.doc]

| **Stimulation with**  **A** | **% of CD4+ T cells producing** | | | | | | |
| --- | --- | --- | --- | --- | --- | --- | --- |
|  | **IFN-γ** | **TNF-α** | **IL-2** | **IL-4** | **IL-17** | **IL-10** | **CD107** |
| **ΤΝF-matured DCs (-)** | 0.02 – 0.21 | 0.02 – 6.23 | 0.04 - 0.16 | 0.00 -0.54 | 0.00 - 0.03 | 0.01 - 0.08 | 1.43 – 15.28 |
| **TNF-matured DCs (+)** | 0.05 - 23.30 | 0.93 - 58.58 | 0.10 - 54.21 | 0.03 - 2.21 | 0.06 - 2.03 | 0.01 - 0.11 | 40.43 - 62.05 |
| **prοΤα-matured DCs (-)** | 0.01 - 0.42 | 0.02 - 3.82 | 0.03 - 0.28 | 0.02 - 0.37 | 0.02 - 0.33 | 0.01 - 0.17 | 0.80 - 5.51 |
| **prοΤα-matured DCs (+)** | 0.11 - 22.07 | 0.57 - 72.76 | 0.17 - 57.44 | 0.02 - 4.42 | 0.04 - 4.74 | 0.01 - 0.20 | 22.20 - 56.10 |
| **prοΤα(100-109)-matured DCs (-)** | 0.01 - 3.87 | 0.03 - 10.39 | 0.02 - 0.71 | 0.01 - 0.49 | 0.00 - 0.14 | 0.01 - 0.20 | 1.82 - 10.86 |
| **prοΤα(100-109)-matured DCs (+)** | 0.07 - 22.93 | 0.24 - 34.72 | 0.08 - 37.18 | 0.00 - 1.64 | 0.02 - 2.31 | 0.01 - 0.23 | 27.89 - 45.90 |

| **Stimulation with** | **% of CD8+ T cells producing** | | | | | | | | |
| --- | --- | --- | --- | --- | --- | --- | --- | --- | --- |
|  | **IFN-γ** | **TNF-α** | **IL-2** | **IL-4** | **IL-17** | **IL-10** | **IL-5** | **IL-13** | **CD107** |
| **ΤΝF-matured DCs (-)** | 0.00 - 1.86 | 0.00 - 4.90 | 0.09 - 2.52 | 0.00 | 0.00 | 0.00 - 0.11 | 0.61 - 3.56 | 2.13 - 11.35 | 0.34 - 3.70 |
| **TNF-matured DCs (+)** | 0.33 - 67.84 | 12.54 - 71.6 | 0.44 -30.10 | 0.40 - 0.97 | 0.00 - 0.07 | 0.00 - 0.19 | 0.66 - 2.07 | 2.55 - 7.41 | 46.60 - 58.23 |
| **prοΤα-matured DCs (-)** | 0.00 - 0.40 | 0.03 - 15.37 | 0.03 - 0.53 | 0.05 - 0.06 | 0.00 - 0.02 | 0.01 - 0.16 | 0.70 - 1.38 | 2.14 - 4.76 | 0.17 - 2.80 |
| **prοΤα-matured DCs (+)** | 0.15 - 64.15 | 7.51 - 64.60 | 0.23 - 26.14 | 0.13 - 2.01 | 0.00 - 0.08 | 0.01 - 0.16 | 0.57 - 1.40 | 3.79 - 11.17 | 36.86 - 62.34 |
| **prοΤα(100-109)-matured DCs (-)** | 0.00 -0.36 | 0.02 - 15.21 | 0.02 - 1.13 | 0.02 - 0.00 | 0.00 - 0.02 | 0.02 - 0.33 | 0.65 - 1.12 | 2.03 - 4.30 | 0.50 - 2.17 |
| **prοΤα(100-109)-matured DCs (+)** | 0.14 - 37.93 | 2.53 - 69.20 | 0.24 - 11.19 | 0.00 | 0.00 | 0.02 - 0.20 | 0.68 - 1.43 | 2.04 - 12.61 | 17.24 - 41.99 |

| **B**  **Stimulation with** | **ratio of CD8+ T cells producing** | |
| --- | --- | --- |
|  | **IFN-γ/IL-5** | **IFN-γ/IL-13** |
| **ΤΝF-matured DCs (-)** | 3.80 - 4.81 | 1.09 - 1.51 |
| **TNF-matured DCs (+)** | 20.06 - 124.11 | 5.60 - 32.12 |
| **prοΤα-matured DCs (-)** | 2.74 - 3.53 | 0.75 - 0.91 |
| **prοΤα-matured DCs (+)** | 31.20 - 147.93 | 3.91 - 22.25 |
| **prοΤα(100-109)-matured DCs (-)** | 2.92 - 5.26 | 0.76 - 1.20 |
| **prοΤα(100-109)-matured DCs (+)** | 40.65 - 118.32 | 4.61 - 39.44 |
