# Supplementary figures and images for: Prothymosin α and a prothymosin α-derived peptide enhance TH1-type immune responses against defined HER-2/neu epitopes
Source: BMC Immunol. 2013 Sep 22;14:43. doi: 10.1186/1471-2172-14-43 (PMC3852324; doi:10.1186/1471-2172-14-43)

## Slide 1
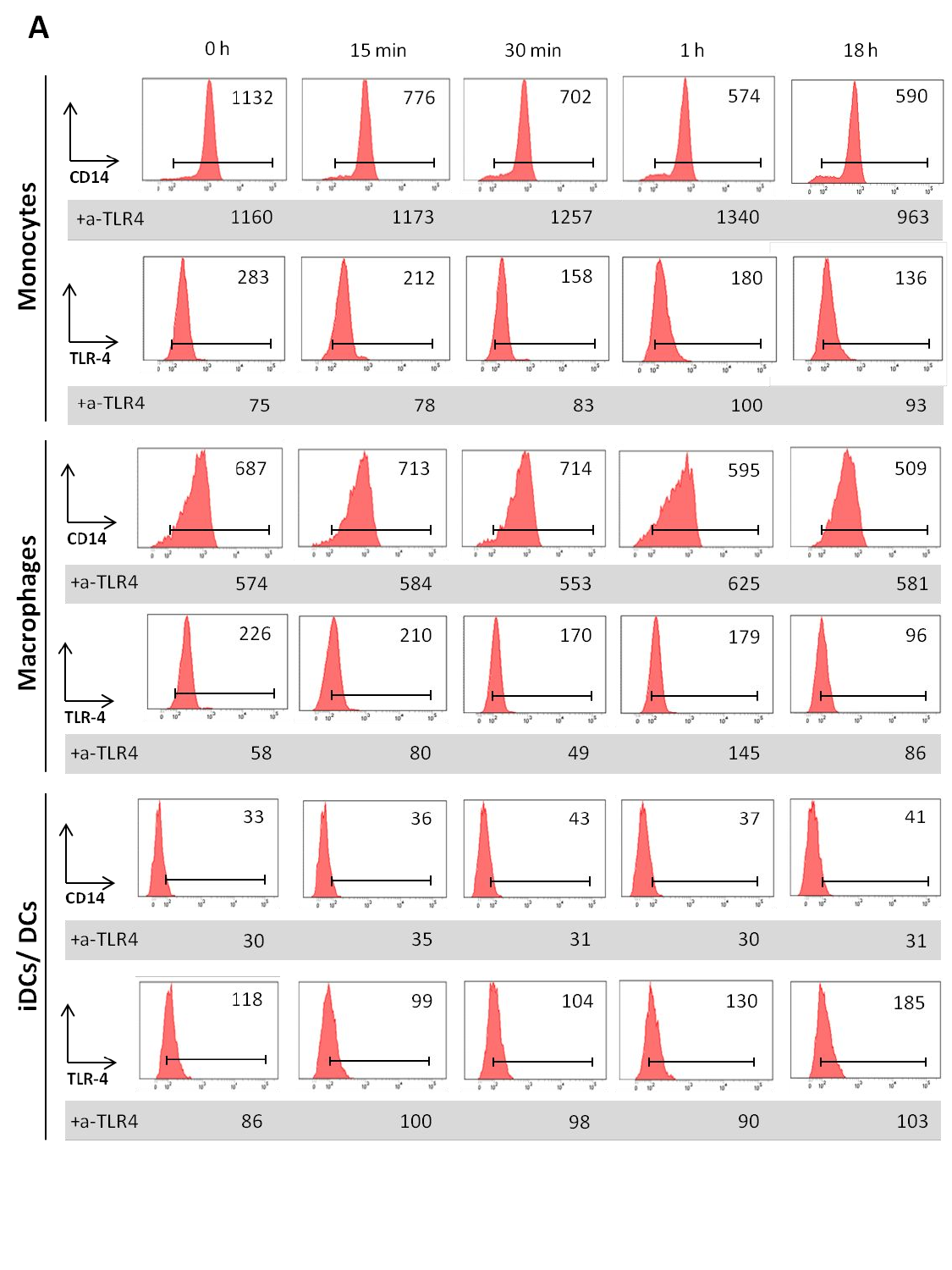

## Slide 2
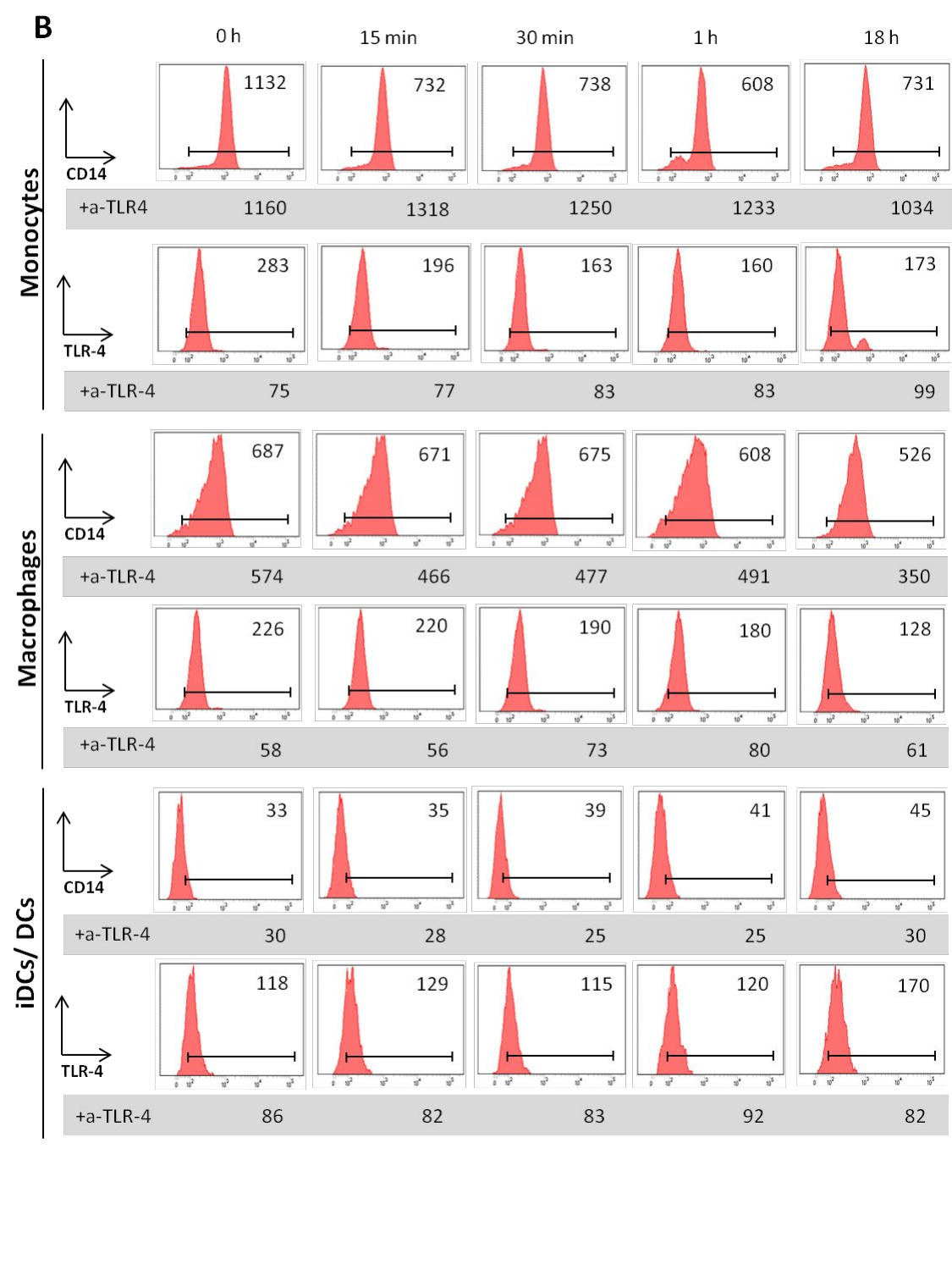

## Slide 3
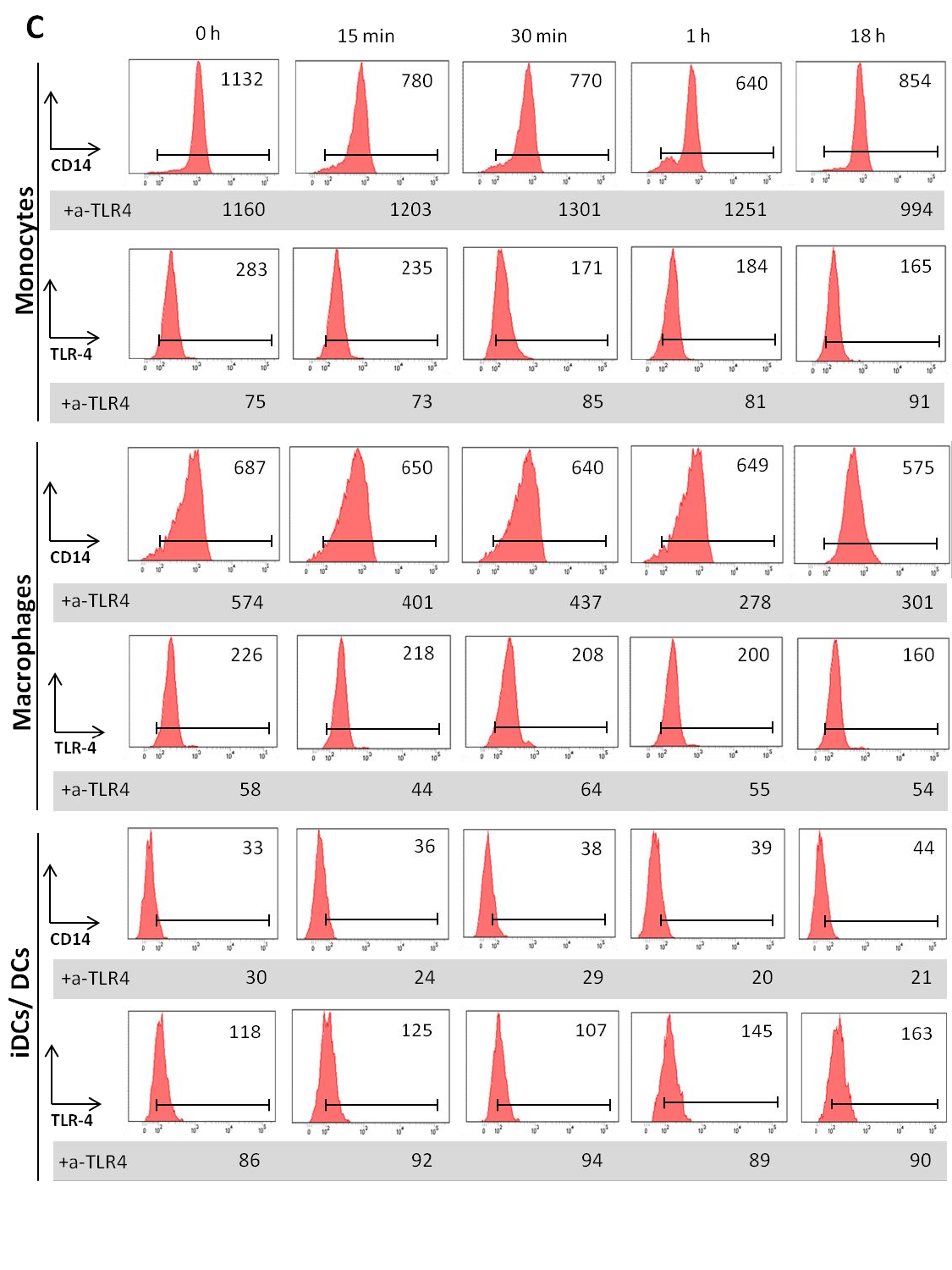

## Slide 4
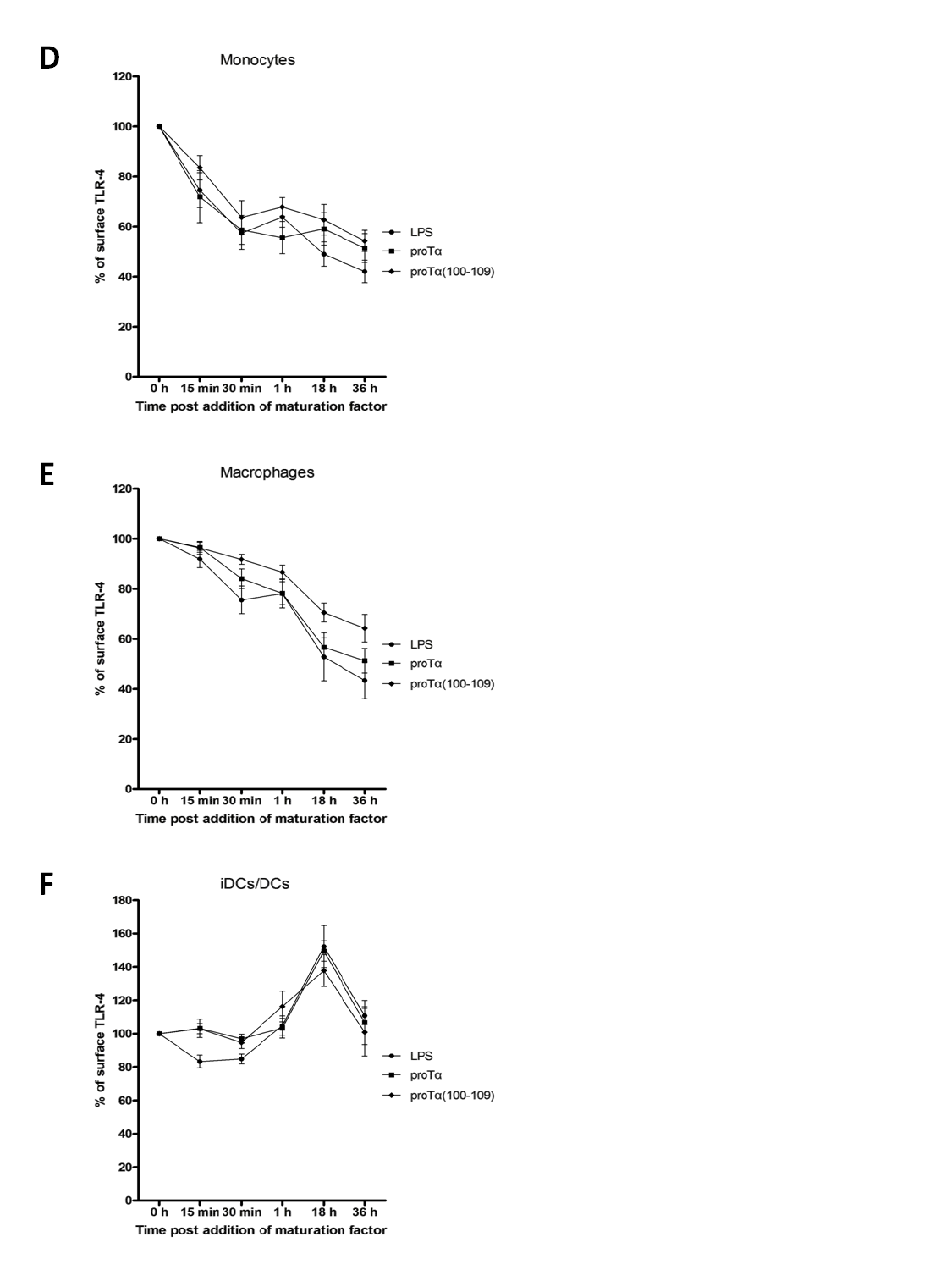

## Slide 5
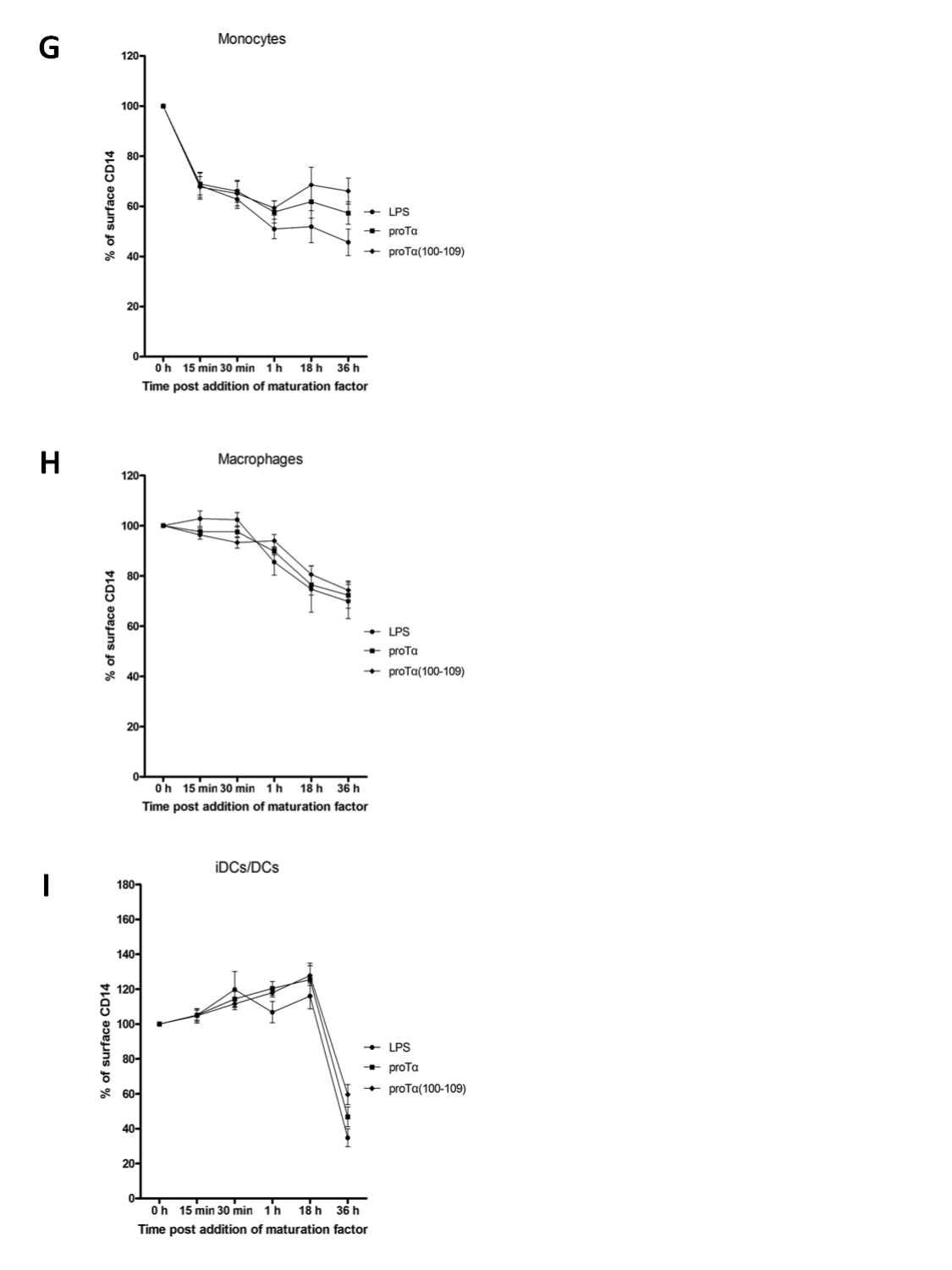

Supplement: Additional file 2: Figure S1 — Kinetics of CD14 and TLR-4 surface expression on monocytes, macrophages and iDCs/DCs upon stimulation with LPS, proTα or proTα(100–109). Monocytes, macrophages and iDCs (0 h) were stimulated with LPS (A), proTα (B), or proTα(100–109) (C) for 15 min, 30 min, 1 h and 18 h and assessed for the surface expression of CD14 and TLR-4 using flow cytometry. MFI values in the presence of neutralizing anti-TLR-4 Ab (+ a-TLR-4) are shown below each histogram. Histograms are from one representative donor of 3 tested. Using the loss of cell surface expression as a readout for TLR-4 and CD14 endocytosis from 0–36 h [31], data from all three donors are shown as mean values ± SDs for TLR-4 (D, E, F) and CD14 (G, H, I). [file 1471-2172-14-43-S2.pptx]

## Slide 1
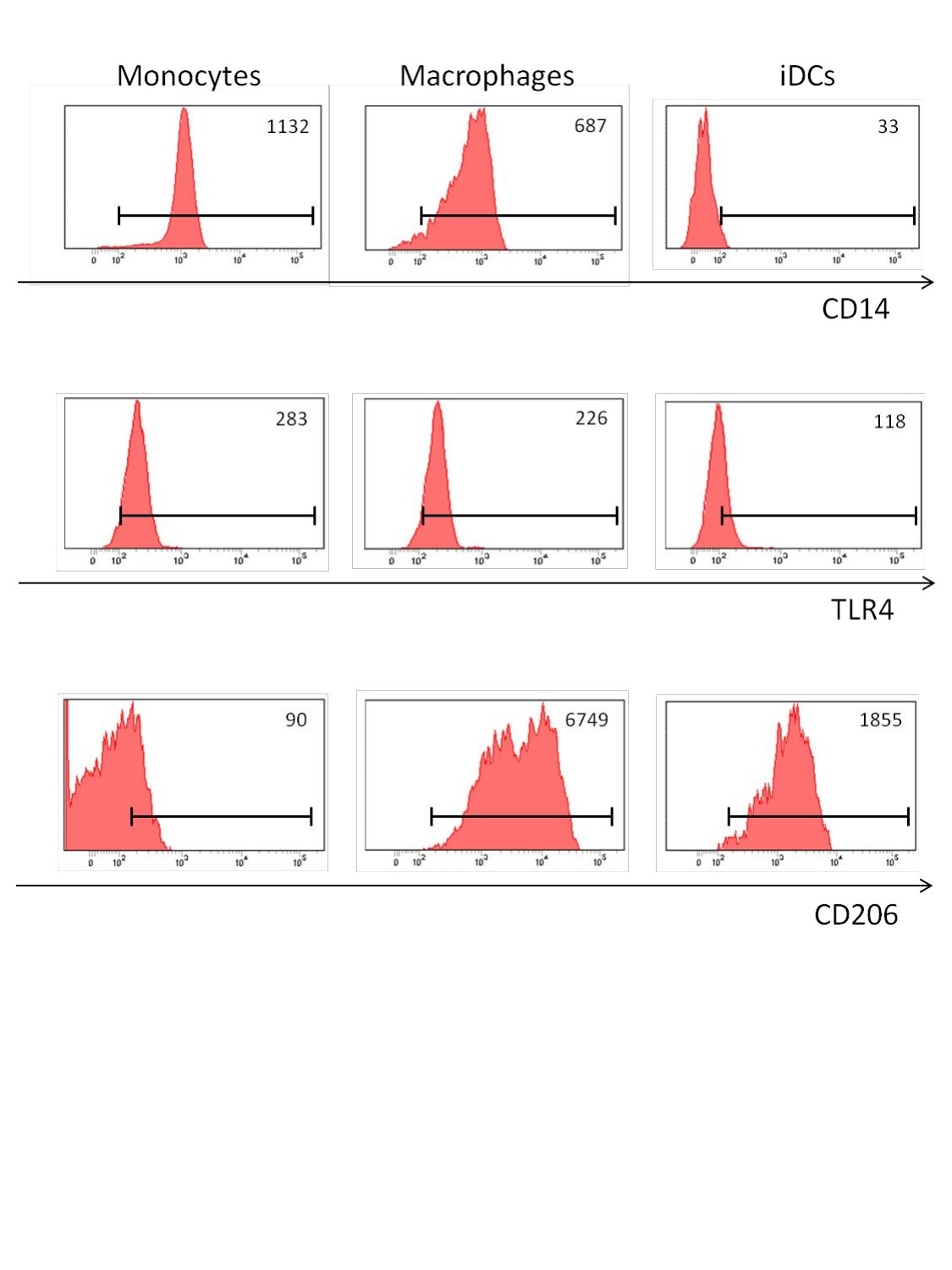

Supplement: Additional file 3: Figure S2 — CD14, TLR-4 and CD206 expression on monocytes, monocyte-derived macrophages and monocyte-derived iDCs. Macrophages were generated from human monocytes upon incubation with 100 ng/mL GM-CSF for 5 days. Human monocytes were isolated and iDCs were generated as described in Methods. Monocytes, macrophages and iDCs were assessed for the surface expression of CD14, TLR-4 and CD206 (as a specific marker for macrophages and DCs), using flow cytometry. Histograms are from one representative donor of 3 tested and numbers indicate MFIs. [file 1471-2172-14-43-S3.pptx]
